# Adverse Childhood Experiences Underlie Race‐Related Differences in Neural Reactivity to Stress

**DOI:** 10.1155/bn/9924677

**Published:** 2026-05-30

**Authors:** Devon K. Grey, Elizabeth S. Davis, Adam M. Goodman, Juliann B. Purcell, Heather E. Dark, Nathaniel G. Harnett, Anudeep Bolaram, Cameryn T. Eickstead, Hayden C. Cook, Sylvie Mrug, David C. Knight

**Affiliations:** ^1^ Department of Psychology, University of Alabama at Birmingham, Birmingham, Alabama, USA, uab.edu; ^2^ Current Institution: Department of Surgery, Boston University School of Medicine, Boston, Massachusetts, USA, bu.edu; ^3^ Current Institution: Division of Depression and Anxiety Disorders, McLean Hospital, Belmont, Massachusetts, USA, mcleanhospital.org; ^4^ Current Institution: Department of Psychiatry, Harvard Medical School, Boston, Massachusetts, USA, harvard.edu

**Keywords:** adverse childhood experiences, neuroimaging, race-related differences, social neuroscience, stress

## Abstract

Black individuals are disproportionately exposed to adverse childhood experiences (ACEs) in comparison to White individuals, including greater violence exposure, neighborhood disadvantage, and poverty. Neural circuitry that includes the amygdala, hippocampus, prefrontal cortex (ventromedial, dorsomedial, and dorsolateral), and inferior parietal lobule support stress‐related emotional processes. ACEs may modify emotion‐related activity within these brain regions, which, in turn, may modulate emotional behavior. However, the extent to which ACEs underlie race‐related differences in emotional function remains to be determined. Therefore, this study investigated whether stress‐elicited brain activity varies with race‐related differences in ACEs. Functional magnetic resonance imaging (fMRI) data from 301 Black and White participants were acquired during the Montreal Imaging Stress Task. Violence exposure, neighborhood disadvantage, and family income were measured during adolescence to assess ACEs, whereas stress‐elicited brain function was assessed in emerging adulthood. Behavioral (stress ratings) and psychophysiological data (skin conductance and heart rate) were collected alongside fMRI. Race‐related differences were observed in behavioral (stress ratings), psychophysiological (heart rate), and neural (fMRI) responses to stress. Further, a significant relationship was observed between stress reactivity and ACEs. Importantly, adjusting for ACEs reduced race‐related differences in stress reactivity (stress ratings and brain function), suggesting that the neurobehavioral response to stress may be shaped, in part, by ACEs. These findings provide new insight into the socioenvironmental factors that influence emotional function.

## 1. Introduction

Systemic racial inequalities within the United States have resulted in the disproportionate exposure of Black individuals to greater violence exposure, neighborhood disadvantage, and poverty than White individuals [1–3]. These types of adverse experiences may be particularly detrimental during adolescence, an important stage of neural and emotional development [4–6]. In fact, previous work has demonstrated a strong connection between adverse childhood experiences (ACEs) and psychopathology [7–9]. Thus, there may be race‐related differences in the expression of psychopathology that stem from race‐related differences in ACEs. However, prior research has repeatedly found less anxiety, depression, and posttraumatic stress in Black than White individuals, often described as the “Black–White Mental Health Paradox” [10, 11]. The seemingly counterintuitive relationship between race and psychopathology may, in fact, be due to greater emotional resilience among Black than White individuals [11, 12]. Stress‐related emotional processes are supported by the amygdala, hippocampus, prefrontal cortex (i.e., ventromedial [vmPFC], dorsomedial [dmPFC], and dorsolateral [dlPFC] prefrontal cortex [PFC]), and inferior parietal lobule (IPL) [13, 14]. Further, ACEs have been linked to differences in the neurobiology of these brain regions [15–17]. Therefore, race‐related differences in ACEs may alter the neurobiological processes that support emotional function. Ultimately, these differences may impact psychological outcomes (e.g., “Black–White Mental Health Paradox”).

Stress‐related emotional processes (e.g., susceptibility or resilience to psychopathology) are supported by the amygdala, hippocampus, PFC (e.g., vmPFC, dmPFC, and dlPFC), and IPL [18–21]. For example, frontoparietal regions (e.g., dlPFC and IPL) coordinate emotion regulation processes (e.g., distraction, suppression, and reappraisal) that modulate susceptibility to anxiety, depression, and posttraumatic stress [22, 23]. Frontoparietal projections through the dmPFC and vmPFC to the amygdala also appear to modulate emotional responses [24–27]. Similarly, projections between the dlPFC and hippocampus support anticipatory processes that are important for the prediction of stressful or threatening events, which also appear to modulate amygdala activity [28–30]. In turn, amygdala projections to the hypothalamus support the peripheral expression of emotion [31, 32]. Collectively, this neural circuitry supports important emotional processes that promote susceptibility and/or resilience to emotional distress [13, 19, 33].

ACEs appear to have lasting effects on the neurobiological processes that support stress‐related emotional function. Specifically, amygdala, hippocampus, vmPFC, dmPFC, dlPFC, and IPL activity appears to vary with ACEs [16, 17]. For example, greater amygdala, hippocampus, vmPFC, and dmPFC activity has been observed in those exposed to violence at an early age [34–37]. Similarly, early exposure to disadvantaged neighborhoods and poverty has been linked to altered stress reactivity within these same regions [16, 17, 38]. In turn, alterations within this neural circuitry have been linked to greater psychopathology [39–41]. Thus, ACEs may be linked to neurobiological alterations that influence susceptibility and/or resilience to psychopathology. Further, given the race‐related disparities in ACEs, there may be race‐related differences in the neurobiological processes that mediate emotional function. Therefore, identifying race‐related differences in stress‐elicited brain function would advance our knowledge of the lasting repercussions of ACEs.

ACEs (e.g., violence exposure, neighborhood disadvantage, and poverty) have been linked to a greater risk of psychopathology [7, 8]. Further, systemic racial inequalities have been linked to greater exposure to ACEs among Black than White individuals [2, 42]. However, Black individuals typically do not report a greater amount of psychopathology in comparison to their White counterparts (e.g., “Black–White Mental Health Paradox”) [10, 43]. The lower rates of psychopathology observed in Black individuals may be due to race‐related differences in emotional resilience to adverse experiences [10, 12, 44]. In fact, prior research suggests that Black individuals may respond to stress differently than White individuals [45, 46]. For example, Black individuals often appraise stressors as less distressing than White individuals who have experienced the same stressor [45, 46]. The lower stress appraisal seen in Black individuals suggests that greater emotional resilience may develop as a result of systemic exposure to racial inequalities. For example, positive self‐concepts and active coping styles (e.g., when confronting stressors) have been linked to greater resilience in children who have experienced adversity [47–49]. Thus, determining the relationship between ACEs and race‐related differences in emotional function may offer new insight into socioenvironmental factors associated with mental health outcomes.

The present study investigated the interrelationships among stress‐elicited brain function, psychopathology (i.e., psychological distress), and ACEs linked to systemic racial inequalities. Specifically, a psychosocial stress task was used to examine race‐related differences in stress‐elicited behavioral, psychophysiological, and neural responses. We then compared these stress‐elicited responses to ACEs. We hypothesized that more ACEs would be reported by Black than White participants and that race‐related differences in stress reactivity would be observed within the amygdala, hippocampus, PFC, and IPL. We also hypothesized that any race‐related differences in stress reactivity would vary with ACEs and that adjusting for ACEs would reduce race‐related differences in stress reactivity. This study investigated race‐related differences in the neurobehavioral response to stress to gain new insights into the long‐term impact that systemic racial inequalities may have on emotional function.

## 2. Materials and Methods

### 2.1. Participants

Participants included 301 volunteers (152 female, 149 male; 201 Black, 100 White; age: 20.03 ± 1.51 years [mean ± SD]; age range: 17–23 years; right‐handed). Volunteers were recruited from the Birmingham‐Hoover, Alabama Metropolitan area from a larger cohort of participants (*N* = 1594) who had previously participated in the Healthy Passages study [50]. Participants were initially interviewed across three waves of data collection (Wave 1: 11.19 ± 0.51, Wave 2: 13.06 ± 0.50, and Wave 3: 16.14 ± 0.54; mean ± SD years of age). A fourth interview was conducted for the present study (Wave 4 age: 19.17 ± 1.17 years), which was followed by a neuroimaging session (age: 20.03 ± 1.51). Data collection began in the fifth grade, and the timing of the four waves of data acquisition was chosen to identify the initiation of risk behaviors, changes in risk behaviors, and potential relationships with critical transition periods (i.e., elementary to middle school and prepubescence to puberty). Written informed consent was provided by each participant as approved by the Institutional Review Board for Human Use (IRB‐120508007) of the University of Alabama at Birmingham.

#### 2.1.1. Sample Size and Statistical Power

Statistical power was calculated for Wave 4 of data collection and the subsequent neuroimaging session using the “test of not‐close fit” (*α* = 0.05, power = 0.80) [51]. Minimum sample sizes of 1175 for Wave 4 and 240 for the neuroimaging session were estimated. The power to detect well‐fitting models was 1 for Wave 4 and 0.82 for the neuroimaging session. Thus, the sample sizes for Wave 4 (*N* = 1594), the neuroimaging session (*N* = 350), and the final neuroimaging sample (i.e., following exclusions; *N* = 301) are sufficient to adequately test the proposed hypotheses.

#### 2.1.2. Exclusion Criteria

Exclusion criteria for neuroimaging recruitment included brain or spinal abnormalities, head injury, blood or circulation disorders, pregnancy, and contraindications for magnetic resonance imaging (MRI). Participants currently taking stimulant, selective serotonin reuptake inhibitor, antipsychotic, benzodiazepine, or beta‐blocker medications were also excluded from participation. Consistent with prior research [52–56], approximately 14% of neuroimaging participants (i.e., 49 of 350 participants) were excluded from subsequent analyses because of concerns with data quality that included image artifacts, excessive movement, and incomplete data.

#### 2.1.3. Demographic Comparisons

Demographic differences were assessed between participants that were and were not excluded (i.e., due to data quality) from the present analyses. No differences were observed in the sex, race, violence exposure, neighborhood disadvantage, or socioeconomic status of participants who were and were not excluded from analyses (*p* < 0.05). Demographic information was also compared between participants from the Healthy Passages study that completed the MRI session and those who did not. There were no differences in sex, violence exposure, neighborhood disadvantage, or socioeconomic status (*p* < 0.05). However, a larger proportion (*χ* ^2^[1] = 12.20, *p* < 0.001) of Black than White volunteers participated in the MRI session. Examination of missing data utilized independent samples *t*‐tests and chi‐square tests to investigate potential differences with ACEs and participant race.

### 2.2. Functional Magnetic Resonance Imaging (fMRI)

Siemens 3 Tesla Allegra (*n* = 251) and Prisma (*n* = 50) MRI scanners were used to collect blood‐oxygenation‐level‐dependent fMRI data (Allegra and Prisma fMRI parameters: repetition time [TR] = 2000 ms, echo time [TE] = 30 ms, flip angle [FA] = 70°, field of view [FOV] = 24 cm, matrix = 64 × 64, slice thickness [ST] = 4 mm). A T1‐weighted MPRAGE (Magnetization Prepared Rapid Gradient Echo) sequence was used to collect an anatomical image (Allegra MPRAGE parameters: TR = 2300 ms, TE = 3.90 ms, FA = 12°, FOV = 25.60 cm, matrix = 256 × 256, ST = 1 mm, gap = 0.50 mm; Prisma MPRAGE parameters: TR = 2300 ms, TE = 2.98 ms, FA = 9°, FOV = 25.60 cm, matrix = 256 × 256, ST = 1 mm, gap = 0.50 mm), which served as an underlay for fMRI data. Analysis of Functional NeuroImages (AFNI) software was used to preprocess and analyze neuroimaging data [57]. The data were slice‐time corrected, registered to the fifth image of the first fMRI scan, then smoothed using a 4‐mm full‐width‐at‐half‐maximum (FWHM) Gaussian kernel. The subject‐level analyses incorporated regressors of no interest to account for visual and auditory feedback, participant behavioral responses (i.e., button presses and joystick movements), and six head motion parameters. Math problems served as the regressor of interest. An instantaneous response function was used to model visual feedback, whereas duration‐modulated hemodynamic response functions served as regressors for math trials and auditory feedback. Further motion correction was completed by excluding high motion images (i.e., > 3% of voxels were outliers). Participants were excluded from further analyses when the number of usable volumes was less than 80% (*n* = 1). The fMRI data were then resampled (1 mm^3^) and normalized (MNI 152) [58].

### 2.3. Montreal Imaging Stress Task

Stress‐elicited neural, behavioral, and psychophysiological responses were assessed using the Montreal Imaging Stress Task (MIST) [59]. Previous research [14, 60] has described the specific adaptation of the MIST used in this study. The MIST was presented using an event‐related design (54 trials) that consisted of separate Control and Stress scans lasting 7 min and 54 s. Each trial (6‐s duration) began with a unique math problem that was presented (0.50–5‐s duration) along with answer options that ranged from 0 to 9. Participants used a joystick to highlight and select their answers (Current Designs; Philadelphia, Pennsylvania, United States). After an answer was selected, a variable duration fixation cross (0.50–5‐s duration) was presented, which was followed by visual feedback (i.e., “Right,” “Wrong,” or “Time Out”) lasting 0.50 s. A fixation cross was presented for 1–3 s during the intertrial interval, which separated each MIST trial.

Participants practiced the MIST before the imaging session by answering a series of math problems. Participant performance on these practice trials was later used to set the task′s difficulty level during neuroimaging. Difficulty levels ranged from easy (add or subtract 2 one‐digit numbers), medium‐easy (add or subtract 3 one‐digit numbers), medium‐hard (add or subtract 4 one or two‐digit numbers), and hard (add, subtract, or multiply 4 one or two‐digit numbers). Participants were unable to consistently answer the medium‐hard and hard difficulty levels within the 5‐s time limit. Therefore, the easy or medium‐easy difficulty levels (based on the participant′s response time) were used during the neuroimaging session. Math problem difficulty was held constant across both conditions of the MIST (Control and Stress MIST).

Investigators told participants not to worry about making mistakes before they began the Control scan in an attempt to lower the participant′s stress level. Participants also received positive auditory feedback at four points during the Control scan (e.g., “You are doing great! Looks like you′ve got this under control. Good job!”). Further, math problems were always presented for 5 s to give participants sufficient time to make their response. Following the Control scan (but before the Stress scan), researchers attempted to raise the participant′s stress level by noting that at least 80% of the math problems must be answered correctly or their data would not be included in the study. Participants also received negative auditory feedback at four points during the Stress scan. The negative auditory feedback was intended to further raise stress levels by noting that participants were not answering enough questions correctly and telling them to try harder. Finally, the amount of time the math problems were presented was adjusted from trial‐to‐trial in a stair‐step manner to ensure participant failure (i.e., only ~50% correct answers). Specifically, the duration of math problems was adjusted in 0.50‐s increments, decreasing the duration by 0.50 s after a correct answer and increasing the duration by 0.50 s after an incorrect answer (range = 0.50–5 s).

### 2.4. Stress Ratings

Stress ratings were collected as a self‐reported measure of stress following the MIST. Participants answered eight questions on a five‐point scale for each MIST condition (“*not at all*” = 1, “*moderately*” = 3, and “*extremely*” = 5). Answers were summed for each MIST condition (Control MIST or Stress MIST) to create an index of stress (range = 8–40). MIST stress ratings show good internal consistency [61]. Stress ratings were not collected from 13 participants. Therefore, stress ratings were analyzed for the 288 participants with complete data. Examination of missing data revealed no significant differences between participants with missing data and participants with complete data for violence exposure (*t*[299] = 1.33, *p* = 0.186), neighborhood disadvantage (*t*[299] = 0.10, *p* = 0.922), family income (*t*[299] = 0.95, *p* = 0.345), or participant race (*χ* ^2^[1] = 0.63, *p* = 0.427).

### 2.5. Heart Rate

Heart rate data were acquired as a physiological measure of stress during each MIST condition. An MRI compatible Siemens photoplethysmograph was used to collect heart rate data (50 Hz) from the left index finger. QRSTool software identified the peak pulse waveforms within the cardiac time series, and CMetX software was used to calculate heart rate (i.e., beats‐per‐minute) for each MIST condition [62]. Heart rate data were unusable for 127 participants due to equipment failure and excessive noise in the signal. Therefore, heart rate was analyzed for the 174 participants with complete data. Examination of missing data revealed no significant differences between participants with missing data and participants with complete data for violence exposure (*t*[231.34] = 1.80, *p* = 0.073; adjusted for unequal variances), neighborhood disadvantage (*t*[299] = 1.14, *p* = 0.255), family income (*t*[299] = 0.96, *p* = 0.339), or participant race (*χ* ^2^[1] = 2.35, *p* = 0.125).

### 2.6. Skin Conductance Response (SCR)

Skin conductance data were recorded to index the peripheral expression of emotion during each MIST condition. A Biopac psychophysiological monitoring system (Goleta, California, United States) monitored skin conductance using methods consistent with prior research [63]. Two disposable (1‐mm diameter) MRI compatible electrodes and isotonic electrode gel were used to measure (10 kHz) skin conductance from the left hand (thenar and hypothenar eminence). Acqknowledge software (4.1.0) was used to low‐pass filter (1 Hz) and down sample (250 Hz) skin conductance data. SCRalyze software (b2.1.8) was then used to bandpass filter (first‐order Butterworth filter; high‐pass cutoff: 0.0159 Hz, low‐pass filter: 1.0 Hz), further down sample (10 Hz), and normalize (z‐transform and mean center) the skin conductance data [64]. The general linear model was used to calculate (beta‐estimates) SCR to math problems, assuming an SCR function without a time or dispersion derivative. SCR data were unusable for 70 participants due to equipment failure (*n* = 7) and low signal‐to‐noise (*n* = 63). Therefore, SCR was analyzed for the 231 participants with complete data. Examination of missing data revealed no significant differences between participants with missing data and participants with complete data for violence exposure (*t*[299] = 0.72, *p* = 0.473), neighborhood disadvantage (*t*[299] = 0.41, *p* = 0.683), or family income (*t*[299] = 1.01, *p* = 0.315). However, missing data did vary with participant race (*χ* ^2^[1] = 5.72, *p* = 0.017), such that Black participants (*n* = 55) had a higher proportion of missing SCR data than White participants (*n* = 15).

### 2.7. Cortisol

Saliva samples were acquired immediately before the fMRI session and again after the MIST was completed to assess the stress‐elicited cortisol response. More specifically, a pretask sample was collected just before the participant entered the scanner (~1 h after arrival). A post‐task sample was then collected after the participant completed the MIST (~20 min after). Saliva samples (2.0 mL) were collected using passive drool and then stored in a −80°C freezer until shipped overnight to the Institute for Interdisciplinary Salivary Bioscience Research (IISBR). The IISBR used a commercial immunoassay to assay samples for cortisol, following the recommended protocol (Salimetrics, LLC, Carlsbad, California). Assays used 25‐*μ*L test volume (sensitivity range = 0.007–3 *μ*g/dL) for singlet determination. Each saliva sample was assayed in duplicate and then averaged before inclusion in subsequent group level analyses (intra‐assay coefficient of variation [COV] < 5%; interassay COV < 15%). The difference score (post‐task − pretask baseline) was used in statistical analyses. Prior studies have restricted the collection of cortisol samples to the afternoon in order to control for diurnal rhythms. We were unable to restrict data collection in this manner due to limitations with participant and MRI scanner availability. Thus, cortisol data were acquired from 8 a.m. to 5 p.m. Outliers (i.e., values 3SD from the mean) in cortisol data were excluded from three participants. Therefore, cortisol was analyzed for the 298 participants with valid data.

### 2.8. Violence Exposure

Violence exposure data (*N* = 301) were acquired during each wave (Waves 1–4) of data collection. Participants answered seven questions at each wave about their exposure to violence over the last year (“*never*” = 0, “*once*” = 1, “*a few times*” = 2, and “*many times*” = 3). Specifically, participants reported how often they witnessed violence (i.e., three questions related to violent threats, physical violence, and threats or actual physical violence with a weapon). Further, participants reported whether they had been victimized by violence (i.e., four questions related to violent threats, physical violence, threats or actual physical violence with a weapon, and violence‐related injuries that required medical attention). Participant responses to the three witnessing questions were averaged for each wave. Similarly, responses to the four victimization questions were averaged for each wave. The individual scales for witnessing and victimization were then combined to create an index of violence exposure for each wave (i.e., witnessing + victimization). Finally, the combined amount of violence exposure from each wave was averaged across Waves 1–4 to create the violence exposure measure used in this study.

### 2.9. Neighborhood Disadvantage

Participant (*N* = 301) residential addresses at Wave 1 (provided by primary caregivers) and block‐level data from the 2000 US census were used to assess neighborhood disadvantage. We used a principal component analysis to derive a factor score based on neighborhood variables that loaded onto a single dimension, which included: % single‐parent families, % adults without a high school diploma, % unemployed adults, % households below the federal poverty line, reverse‐coded % owner‐occupied housing units, and reverse‐coded median household income. The absolute value of all variables loading on this dimension ranged from 0.71 to 0.93. Residential addresses were collected at the initial Wave 1 assessment but were not systematically collected during subsequent waves, limiting our ability to assess neighborhood disadvantage across waves. However, prior research suggests that there is limited movement between disadvantaged and advantaged neighborhoods over time [65–67].

### 2.10. Family Income

Family income was used as an index of socioeconomic status. Family income and household size data were collected from primary caregivers at Waves 1–3, when participants were 11–16 years of age, and living with a parent, guardian, or other caregiver. Family income data were converted to a % of the federal poverty level based on the size of the family′s household. Family income data were then averaged across Waves 1–3 to create an index of socioeconomic status across adolescence. Family income was not collected during Wave 4, because participants were ~19 years of age and in a transitional stage of emerging adulthood (e.g., obtaining jobs, moving away from home, attending college, and getting married). Income during this transitional period would not provide a comparable index of socioeconomic status relative to household‐level caregiver income. Missing income data (*n* = 14) were previously imputed [68] using SAS (Version 9.4, PROC MI). Therefore, family income was analyzed for all participants (*N* = 301).

### 2.11. Depression Symptoms

Symptoms of depression were assessed during Wave 4 using the Diagnostic Interview Schedule for Children (DISC) [69]. The Major Depression Subscale of the DISC was used to measure depression over the last year. Participants (*N* = 301) answered six questions as “no” or “yes” (no = 0, yes = 1). Participant responses were summed to index depressive symptoms (range = 0–6). The Major Depression Subscale demonstrates good to excellent test–retest reliability [70, 71].

### 2.12. Anxiety Symptoms

Symptoms of anxiety were assessed during Wave 4 using the Revised Children′s Manifest Anxiety Scale (RCMAS) [72]. The Physiological Anxiety subscale of the RCMAS measured anxiety across the lifetime. Participants (*N* = 301) answered 10 questions as “no” or “yes” (no = 0, yes = 1). Participant responses were summed to index anxiety symptoms (range = 0–10). The RCMAS demonstrates good to excellent test–retest reliability [73, 74].

### 2.13. Posttraumatic Stress Symptoms

Symptoms of posttraumatic stress were assessed at Wave 4 using the Childhood Posttraumatic Stress Disorder Symptom Scale (CPSS) [75]. The CPSS measured posttraumatic stress over the past 2 weeks. Participants answered 17 questions on a four‐point scale (“*not at all/only once*” = 0, “*once per week or less/once in a while*” = 1, “*two to four times per week/half the time*” = 2, and “*five or more times per week/almost always*” = 3). Participant responses were summed to index posttraumatic stress symptoms (range = 0–51). The CPSS demonstrates excellent test–retest reliability [76, 77]. Posttraumatic stress data were not available for nine participants. Therefore, analyses included the 292 participants with complete data. Examination of missing data revealed no significant differences between participants with missing data and participants with complete data for violence exposure (*t*[299] = 1.31, *p* = 0.192), neighborhood disadvantage (*t*[299] = 0.28, *p* = 0.781), family income (*t*[299] = 0.66, *p* = 0.509), or participant race (*χ* ^2^[1] = 2.04, *p* = 0.153).

## 3. Analyses

### 3.1. Race‐Related Differences in Participant Characteristics

Independent samples *t*‐tests (two‐tailed) assessed race‐related differences in demographic data. Specifically, *t*‐tests assessed race‐related differences in participant age at the MRI session, ACEs, and psychological distress. Race‐related differences in the proportion of female and male participants were assessed using one‐sample proportion tests (score test).

### 3.2. Stress Measures

Group differences in stress ratings, heart rate, and SCR were assessed using ANCOVA. The analysis included a within‐subjects factor for MIST condition (Control or Stress MIST), between‐subjects factor for race (Black or White), and covariate for scanner (Allegra or Prisma). Group differences in cortisol levels were also assessed using ANCOVA that included a within‐subjects factor for task (pretask baseline or post‐task), between‐subjects factor for race (Black or White), and covariate for scanner (Allegra or Prisma).

### 3.3. Neural Reactivity to Stress

Linear mixed effects (LME) analyses (AFNI: 3dLME) of the neuroimaging data were used to identify race‐related differences in neural reactivity to stress (i.e., Control vs. Stress MIST). Covariates included scanner type (i.e., Allegra and Prisma) and participant sex (i.e., female and male). The mean signal response from areas of activation was then extracted (AFNI: 3dROIstats) to further describe the relationship between race and neural reactivity to stress. Neuroimaging results were visualized using AFNI [57] and Surf Ice software [78].

**Figure 1:**
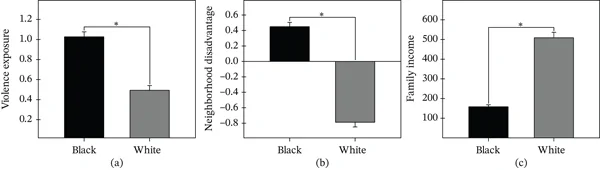
Racial differences in ACEs. Note. ACEs = adverse childhood experiences. Black participants reported higher levels of (a) violence exposure, (b) greater neighborhood disadvantage, and (c) lower family income than White participants. Error bars = standard error of the mean. ∗*p* < 0.001.

Extracted signal responses from the LME analysis were used in post hoc independent samples *t*‐tests (two‐tailed) to identify patterns of activation uniquely associated with Black and White participants. Linear regressions then assessed whether the observed stress‐elicited brain activity (i.e., race‐related differences) from a priori regions of interest varied with ACEs. Specifically, linear regression models compared neural reactivity to stress (Stress − Control MIST; dependent variable) to the three ACEs (i.e., violence exposure, neighborhood disadvantage, and family income; independent variables). Covariates for scanner type and sex were included in these analyses. However, a covariate for race was not included to avoid potential multicollinearity issues (i.e., due to race‐related differences in ACEs [Table 1; Figure 1]) and to complete subsequent comparative analyses of race‐related differences that consider ACEs. The full model included scanner, sex, and all three ACEs (i.e., violence exposure, neighborhood disadvantage, and family income).

**Table 1:** Participant characteristics.

	Black		White		*p* value
Sex (female)	108 (71.05%)		44 (28.95%)		< 0.001
Sex (male)	93 (62.42%)		56 (37.58%)		0.002
	Mean	SD	Mean	SD	*p* value
Scan age (years)	20.21	1.55	19.67	1.38	0.003
Violence exposure	1.03	0.70	0.49	0.47	< 0.001^ab^
Neighborhood disadvantage	0.45	0.80	−0.79	0.61	< 0.001^ab^
Family income	157.89	143.37	508.85	280.73	< 0.001^ab^
Depression	1.85	1.54	2.16	1.69	0.107
Anxiety	2.57	2.03	3.03	2.10	0.070
Posttraumatic stress	8.46	10.27	6.61	7.44	0.079^a^

*Note:* The values for sex reflect the number of Black (*n* = 201) and White (*n* = 100) participants that are female or male. % values represent the proportion of females who are Black or White, and males who are Black or White. The *p* value for sex represents differences in the proportion of race (i.e., Black or White) for either females or males. *p* values for scan age, violence exposure, neighborhood disadvantage, family income, depression, anxiety, and posttraumatic stress represent the difference between Black and White participant groups. Multiple comparison corrections were completed using the Bonferroni correction for the three ACEs (0.05/3 = 0.017).

Abbreviation: SD = standard deviation.

^a^Adjusted for unequal variances.

^b^Indicates the results survived Bonferroni correction.

#### 3.3.1. Neuroimaging Cluster Thresholding

A mask was created to restrict voxel‐wise neuroimaging analyses to the gray matter of the brain. Specifically, the mask was created by averaging each participant′s anatomical image (MPRAGE) and then segmenting the averaged image into different tissue compartments (AFNI: 3dSeg). Subsequent analyses of fMRI data were limited to the gray matter of the brain using the segmented mask. AFNI′s 3dClustSim (version compiled July 9, 2016) was used to complete Monte Carlo simulations, which were used to determine the voxel‐wise family‐wise error (FWE) corrected significance threshold (*a* < 0.05). Smoothness values for the simulations were determined based on the mixed model (spatial) autocorrelation function (‐acf option) and FWHM (AFNI: 3dFWHMx) parameters obtained from the residuals of subject‐level analyses. The simulation resulted in a 467‐mm^3^ critical cluster extent volume threshold, using an uncorrected *p* < 0.005 significance threshold. Separate simulations were then completed to determine small‐volume corrections for the amygdala and hippocampus, resulting in cluster volume thresholds of 90 (amygdala) and 174 mm^3^ (hippocampus).

#### 3.3.2. ACEs Underlie Race‐Related Differences in Neural Reactivity to Stress

##### 3.3.3. Adjusting for ACEs

Effect sizes (i.e., Cohen′s *d*) were used to assess race‐related differences in the “raw” and “residual” neural reactivity to stress. The “raw” model only included covariates for scanner and sex, whereas the “residual” model included covariates for scanner, sex, and ACEs. Effect size analyses utilized the differential fMRI signal response (i.e., Stress − Control MIST) obtained from the original analysis that compared MIST conditions for all participants. Specifically, AFNI′s 3dttest++ (one‐sample *t*‐test) was used to extract the remaining voxel‐wise signal from the differential fMRI signal response for the “raw” and “residual” models.

#### 3.3.4. Effect Size Calculation

The fMRI signal response to stress for the “raw” and “residual” models was used to calculate separate means and standard deviations for Black and White participants (AFNI: 3dMean). These values were used to calculate effect sizes (Cohen′s *d*). These effect sizes were then used to evaluate race‐related differences in “raw” and “residual” neural reactivity to stress (AFNI: 3dcalc). Positive Cohen′s *d* values reflect neural reactivity to stress that was greater in White than Black participants (i.e., White > Black), whereas negative Cohen′s *d* values reflect neural reactivity to stress that was greater in Black than White participants (i.e., Black > White). The effect size estimates for each model (“raw” and “residual”) were then masked to restrict the results to brain regions showing a significant effect for race (LME model) and for which a priori hypotheses had been formed (i.e., amygdala, hippocampus, PFC, and IPL).

#### 3.3.5. Effect Size Confidence Intervals (CIs)

Ninety‐five percent confidence intervals (95% CIs) from the “raw” and “residual” models were calculated to assess whether the effect sizes for each model confidently excluded zero (i.e., the 95% CIs did not cross zero; AFNI: 3dcalc). CIs that excluded zero reflected significant race‐related differences in stress‐elicited brain activity (i.e., results were different from zero), whereas CIs that included zero reflected no race‐related differences in stress‐elicited brain activity (i.e., results were not different from zero). Specifically, the standard error of the Cohen′s *d* values (i.e., from the previous step) was calculated for the “raw” and “residual” models, which was then used to calculate the margin of error (critical value of 1.96; 95% CI). The margin of error was then either added or subtracted from Cohen′s *d* values to calculate the upper and lower CIs for the “raw” and “residual” models. The upper and lower CI values were extracted (AFNI: 3dROIstats) and compared to determine whether they crossed zero for each model.

95% CIs were also independently calculated for positive (i.e., White > Black activity) and negative (i.e., Black > White activity) effect sizes (Cohen′s *d*) to assess whether the effects confidently excluded zero (AFNI: 3dcalc). Specifically, effect sizes from the “raw” and “residual” models were separated into datasets that only contained positive or negative values. The standard error of positive and negative effect sizes was then calculated for each model (“raw” and “residual”) and used to determine the margin of error (critical value of 1.96; 95% CI). The margin of error was then subtracted from the positive Cohen′s *d* values to calculate the lower CIs for the “raw” and “residual” models, and the margin of error was added to the negative Cohen′s *d* values to calculate the upper CIs for the “raw” and “residual” models. Specifically, if the lower CI values for the “positive relationship” (i.e., White > Black) were negative (below zero) or the upper CI values for the “negative relationship” (i.e., Black > White) were positive (above zero), then the CIs crossed zero. Similar to the previous analysis, CIs that excluded zero reflected significant race‐related differences (i.e., results were different from zero), whereas CIs that included zero reflected no race‐related differences in stress‐elicited brain activity (i.e., results were not different from zero).

### 3.4. ACEs Mediate Race‐Related Differences in Stress Reactivity

Parallel mediation models assessed whether the relationship between participant race and stress reactivity (i.e., stress ratings or neural reactivity to stress) was mediated by the ACEs assessed (i.e., violence exposure, neighborhood disadvantage, and family income) (PROCESS Macro) [79, 80]. Mediation models were constructed with participant race as the independent variable, stress reactivity as the dependent variable, and ACEs (i.e., violence exposure, neighborhood disadvantage, and family income) as the mediators. All three ACEs were included as separate regressors in the same model. Neural mediation models used the extracted mean signal response (AFNI: 3dROIstats) from each significant cluster of activation identified by the initial LME analysis within a priori brain regions of interest [57]. The model with stress ratings included a covariate for sex, whereas the models with neural reactivity included covariates for scanner and sex. Mediation models were considered statistically significant if the 95% CI did not cross zero when conducting bootstrapping with a resampling size (*k*) of 5000 [79]. Partial mediation was assumed if the direct effect (i.e., race to stress reactivity) remained significant after mediation, and full mediation was assumed if the direct effect did not remain significant after mediation [79].

## 4. Results

### 4.1. Participant Demographics

Demographic characteristics of study participants are reported in Table 1. Black participants (20.21 ± 1.55 years; mean ± SD) were about 6 months older than White participants (19.67 ± 1.38 years). A larger proportion of Black (*n* = 201) than White (*n* = 100) individuals participated in the study (*z* = 5.82, *p* < 0.001). A similar pattern was observed among both female and male participants. Specifically, there was a larger proportion of female participants who were Black (*n* = 108) than White (*n* = 44) (*z* = 5.19, *p* < 0.001), and a larger proportion of male participants who were Black (*n* = 93) than White (*n* = 56) (*z* = 3.03, *p* = 0.002).

### 4.2. Participant ACEs

The present study identified race‐related differences in ACEs (Table 1; Figure 1). Specifically, higher levels of violence exposure (~2.10 times higher) (*t*[271.31] = 7.84, *p* < 0.001; adjusted for unequal variances), greater neighborhood disadvantage (~2.75 times higher) (*t*[249.56] = 14.90, *p* < 0.001; adjusted for unequal variances), and lower family income (~3.22 times lower) (*t*[125.32] = −11.76, *p* < 0.001; adjusted for unequal variances) were reported by Black than White participants (Table 1). Correlation analyses (Table 2) demonstrated that violence exposure varied negatively with stress ratings (*r* = −0.26, *p* < 0.001) and family income (*r* = −0.32, *p* < 0.001) but varied positively with neighborhood disadvantage (*r* = 0.27, *p* < 0.001). Similarly, neighborhood disadvantage varied negatively with stress ratings (*r* = −0.31, *p* < 0.001) and family income (*r* = −0.59, *p* < 0.001), but varied positively with SCR (*r* = 0.14, *p* = 0.040). Further, family income varied positively with stress ratings (*r* = 0.33, *p* < 0.001).

**Table 2:** Correlations for emotional response and ACEs.

	1	2	3	4	5	6	7	8	9	10
1. SR	1.00	—	—	—	—	—	—	—	—	—
2. HR	0.29∗∗∗	1.00	—	—	—	—	—	—	—	—
3. SCR	0.19∗∗	0.16	1.00	—	—	—	—	—	—	—
4. Cortisol	0.08	−0.07	−0.14∗	1.00	—	—	—	—	—	—
5. VE	−0.26∗∗∗	−0.06	−0.05	0.00	1.00	—	—	—	—	—
6. ND	−0.31∗∗∗	−0.08	0.14∗	−0.05	0.27∗∗∗	1.00	—	—	—	—
7. Income	0.33∗∗∗	0.03	−0.05	0.04	−0.32∗∗∗	−0.59∗∗∗	1.00	—	—	—
8. Depression	0.08	−0.02	−0.07	0.02	0.12∗	−0.08	0.07	1.00	—	—
9. Anxiety	0.07	−0.03	0.02	0.03	0.21∗∗∗	−0.04	0.03	0.53∗∗∗	1.00	—
10. PTS	−0.09	−0.08	0.00	0.03	0.33∗∗∗	0.09	−0.11	0.38∗∗∗	0.50∗∗∗	1.00

Abbreviations: ACE (values reflect Pearson correlation coefficients), adverse childhood experiences; HR, heart rate; income, family income; ND, neighborhood disadvantage; PTS, posttraumatic stress symptoms; SCR, skin conductance response; SR, stress rating; VE, violence exposure.

Significance: ∗*p* < 0.05; ∗∗*p* < 0.01; ∗∗∗*p* < 0.001.

### 4.3. Stress Measures

Correlation analyses (Table 2) revealed that stress ratings varied positively with heart rate (*r* = 0.29, *p* < 0.001) and SCR (*r* = 0.19, *p* = 0.004), but not cortisol (*p* > 0.05). However, SCR varied negatively with cortisol (*r* = −0.14, *p* = 0.035). ANCOVA revealed a main effect of MIST condition (i.e., Control vs. Stress) for stress ratings (*F*[1, 285] = 437.90, *p* < 0.001), heart rate (*F*[1, 171] = 104.07, *p* < 0.001), SCR (*F*[1, 228] = 81.42, *p* < 0.001), and cortisol (*F*[1, 295] = 20.11, *p* < 0.001). Post hoc analyses revealed higher stress ratings for the Stress than Control condition (*t*[287] = 22.02, *p* < 0.001] (Table 3). Similarly, the psychophysiological data showed that heart rate (*t*[173] = 9.32, *p* < 0.001) and SCR (*t*[230] = 10.31, *p* < 0.001) were greater during the Stress than Control condition (Table 3). In contrast, post hoc analyses revealed higher cortisol values for the Control than Stress condition (*t*[297] = −5.83, *p* < 0.001) (Table 3). However, there was no main effect of race for stress ratings, heart rate, SCR, or cortisol (*p* > 0.05).

**Table 3:** Emotional response to Control versus Stress MIST.

	Stress	Control	DF	*t*	*p* value
Stress ratings	25.78 (6.79)	15.21 (5.85)	287	22.02	< 0.001^a^
Heart rate	73.30 (13.23)	68.22 (10.84)	173	9.32	< 0.001^a^
SCR	0.81 (0.69)	0.39 (0.51)	230	10.31	< 0.001^a^
Cortisol	0.19 (0.13)	0.22 (0.16)	297	−5.83	< 0.001^a^

*Note*: The values for Stress and Control reflect the mean (standard deviation). Heart rate (beats per minute). Skin conductance response (beta values). Cortisol (micrograms per deciliter; *μ*g/dL). Multiple comparison corrections were completed using the Bonferroni correction for the four stress measures (0.05/4 = 0.013).

Abbreviations: DF = degrees of freedom; MIST = Montreal Imaging Stress Task; SCR = skin conductance response; *t* = *t*‐statistic.

^a^Indicates the results survived Bonferroni correction.

An interaction effect was observed between MIST condition (i.e., Control vs. Stress) and race for stress ratings (*F*[1, 285] = 22.94, *p* < 0.001) and heart rate (*F*[1, 171] = 5.25, *p* = 0.023), but not SCR or cortisol (*p* > 0.05). Post hoc analyses evaluated stress rating and heart rate differences to the MIST (i.e., Control vs. Stress) for Black and White participants. Both Black and White participants showed larger stress ratings and heart rate to the Stress than Control condition (Table 4). Post hoc contrasts then evaluated the difference in stress ratings and heart rate (i.e., Stress − Control MIST) between Black and White participants (Table 5). White participants showed a greater difference in both stress ratings and heart rate than Black participants (Table 5).

**Table 4:** Response to Control versus Stress MIST between Black and White participants.

	Race	Stress	Control	DF	*t*	*p* value
Stress ratings	Black	24.83 (7.02)	15.82 (6.27)	190	14.95	< 0.001
	White	27.67 (5.91)	14.02 (4.72)	96	19.65	< 0.001
Heart rate	Black	72.20 (13.07)	68.09 (11.60)	109	7.90	< 0.001
	White	75.21 (13.38)	68.43 (9.47)	63	5.83	< 0.001

*Note*: The values for Stress and Control reflect the mean (standard deviation). Significant interactions between MIST condition (i.e., Control vs. Stress) and race were not observed for skin conductance response or cortisol.

Abbreviations: DF = degrees of freedom; MIST = Montreal Imaging Stress Task; *t* = t‐statistic.

**Table 5:** Race‐related differences in stress‐elicited behavioral and physiological responses.

	Black	White	DF	*t*	*p*value
Stress ratings	9.01 (8.33)	13.65 (6.84)	229.31^a^	−5.04	< 0.001
Heart rate	4.10 (5.44)	6.78 (9.30)	88.59^a^	−2.10	0.038

*Note:* The values for Black and White reflect the mean (standard deviation). Stress ratings and heart rate reflect the differential response (i.e., Stress − Control) to the MIST. Significant interactions between MIST condition (i.e., Control vs. Stress) and race were not observed for skin conductance response or cortisol.

Abbreviations: DF = degrees of freedom; MIST = Montreal Imaging Stress Task; *t* = t‐statistic.

^a^Adjusted for unequal variances.

### 4.4. Relationship Between Stress Ratings and ACEs

Differential stress ratings (i.e., Stress − Control MIST) were correlated with ACEs (Table 2). Therefore, secondary correlations were conducted to evaluate whether ACEs were independently related to stress ratings from the Control or Stress MIST condition (Table 6). Stress ratings from the Control condition varied positively with violence exposure (*r* = 0.16, *p* = 0.008) and neighborhood disadvantage (*r* = 0.18, *p* = 0.003), but varied negatively with family income (*r* = −0.19, *p* < 0.001). In contrast, stress ratings from the Stress condition varied negatively with violence exposure (*r* = −0.18, *p* = 0.002) and neighborhood disadvantage (*r* = −0.22, *p* < 0.001), but varied positively with family income (*r* = 0.23, *p* < 0.001).

**Table 6:** Correlations between ACEs and MIST stress ratings.

	VE	ND	Income	SR Control	SR Stress
VE	1.00	—	—	—	—
ND	0.27 ∗∗∗	1.00	—	—	—
Income	−0.32 ∗∗∗	−0.59 ∗∗∗	1.00	—	—
SR Control	0.16 ∗∗	0.18 ∗∗	−0.19 ∗∗∗	1.00	—
SR Stress	−0.18 ∗∗	−0.22 ∗∗∗	0.23 ∗∗∗	0.18 ∗∗	1.00

Abbreviations: ACEs (values reflect Pearson correlation coefficients), adverse childhood experiences; income, family income; MIST, Montreal Imaging Stress Task; ND, neighborhood disadvantage; SR Control, stress ratings for the Control MIST condition; SR Stress, stress ratings for the Stress MIST condition; VE, violence exposure.

Significance: ∗*p* < 0.05; ∗∗*p* < 0.01; ∗∗∗*p* < 0.001.

### 4.5. ACEs Attenuate Race‐Related Differences in Stress Ratings

Post hoc contrasts identified race‐related differences (i.e., Black vs. White) in stress ratings (Table 5). Additionally, stress ratings varied with participant exposure to ACEs (Tables 2 and 6). Therefore, we investigated whether adjusting for ACEs would attenuate race‐related differences in stress ratings. Standardized residuals were saved from linear regression analyses that included differential stress ratings (i.e., Stress − Control MIST) as the dependent variable. The “raw” model only included a covariate for sex, whereas the “residual” model included covariates for sex and each ACE (regression results presented in Table 7). The residual model included separate regressors for violence exposure, neighborhood disadvantage, and family income. The three ACEs were included in the same model because differences were observed between Black and White participants for all three ACEs (Table 1). Similar analyses were not conducted for the other stress measures (i.e., heart rate, SCR, or cortisol) because we did not observe race‐related differences (i.e., SCR and cortisol) and/or relationships with ACEs for these variables (i.e., heart rate and cortisol; Table 2).

**Table 7:** Stress ratings and ACEs.

	*B*	SE	*β*	95% CI	*p* value
VE^a^	−1.71	0.72	−0.14	−3.13, −0.30	0.018
ND^a^	−1.37	0.59	−0.16	−2.53, −0.22	0.020
Income^a^	0.01	0.00	0.19	0.00, 0.01	0.006

*Note:* Stress ratings reflect the differential response (i.e., Stress − Control) to the MIST. The regression model included all three ACEs in the same model (i.e., violence exposure, neighborhood disadvantage, and family income) and a covariate for sex.

Abbreviations: ACEs = adverse childhood experiences; *B* = unstandardized beta coefficient; Income = family income; MIST = Montreal Imaging Stress Task; ND = neighborhood disadvantage; SE = standard error; VE = violence exposure; *β* = standardized beta coefficient; 95% CI = 95% confidence interval.

^a^Significant regression model.

### 4.6. Effect Size Comparison

Race‐related differences in “raw” (i.e., only adjusting for the covariate of sex) and “residual” (i.e., adjusting for covariates of sex and ACEs) stress ratings were assessed using effect sizes (Cohen′s *d*). Commonly accepted Cohen′s *d* guidelines were used to interpret effect sizes [81]. Race‐related differences in stress ratings showed moderate‐to‐high effect sizes before adjusting for ACEs (“raw” model) (*d* = 0.60, 95% CI [0.35, 0.85]; Table 8). However, race‐related differences in stress ratings were reduced to small effect sizes (*d* = 0.02, 95% CI [−0.26, 0.23]; Table 8) after ACEs were included in the model (“residual” model). Further, the 95% CI crossed zero after ACEs were included in the model, suggesting that these effects were no different from zero.

**Table 8:** Stress rating Cohen′s *d* effect sizes and *t*‐test before and after adjusting for ACEs.

Model	Cohen′s *d*	95% CI	DF	*t*	*p*value
Raw	0.60	0.35, 0.85	228.52^a^	−5.10	< 0.001
Residual	0.02	−0.26, 0.23	286	0.14	0.888

*Note:* Effect sizes (Cohen′s *d*) were calculated to assess race‐related differences in “raw” and “residual” stress ratings. The “raw” model only included a covariate for sex, whereas the “residual” model included covariates for sex and ACEs. The left side of the table shows Cohen′s *d* (absolute value) and Cohen′s *d* 95% confidence interval (95% CI) values. The right side of the table represents an independent samples *t*‐test assessing race‐related differences (i.e., Black vs. White) in stress ratings for the “raw” and “residual” models.

Abbreviations: ACEs = adverse childhood experiences; DF = degrees of freedom; MIST = Montreal Imaging Stress Task; *t* = t‐statistic.

^a^Adjusted for unequal variances.

### 4.7. Psychological Distress

We did not observe any race‐related differences (i.e., Black vs. White) in psychological distress symptoms (Table 1). Specifically, anxiety (*t*[299] = −1.82, *p* > 0.05), depression (*t*[299] = −1.62, *p* > 0.05), and posttraumatic stress (*t*[257.65] = 1.77, *p* > 0.05; adjusted for unequal variance) did not differ by race (Table 1). However, correlation analyses identified significant relationships among the three symptoms (Table 2). Specifically, depression varied positively with anxiety (*r* = 0.53, *p* < 0.001) and posttraumatic stress (*r* = 0.38, *p* < 0.001), and anxiety varied positively with posttraumatic stress (*r* = 0.50, *p* < 0.001).

Correlation analyses also revealed significant relationships between the three psychological distress symptoms and violence exposure (Table 2). Specifically, anxiety (*r* = 0.21, *p* < 0.001), depression (*r* = 0.12, *p* = 0.044), and posttraumatic stress (*r* = 0.33, *p* < 0.001) varied positively with violence exposure. However, psychological distress did not vary with stress ratings, heart rate, SCR, cortisol, neighborhood disadvantage, or family income (Table 2; *p* > 0.05). We did not assess whether adjusting for ACEs attenuated the race‐related differences in psychological distress, due to the lack of significant differences between Black and White participants (Table 1).

### 4.8. Neural Reactivity to Stress

#### 4.8.1. Race‐Related Differences in Neural Reactivity to Stress

The LME model revealed neural reactivity to stress (i.e., Control vs. Stress MIST) that varied with participant race (Table 9). Whole‐brain analyses revealed significant activation within a priori regions of interest (i.e., dmPFC, dlPFC, and IPL; Table 9). Whole‐brain analyses also revealed additional activations within the superior parietal lobe and middle temporal, middle occipital, and lingual gyri (Table 9). No significant effects were observed with small‐volume corrections within the amygdala or hippocampus (Table 9).

**Table 9:** Regions showing race‐related differences in neural reactivity to stress.

Region	Hemi	Volume (mm^3^)	x	y	z	F‐stat
dlPFC	Left	2269	−29	−19	64	22.27
dlPFC	Right	723	41	15	49	15.85
dmPFC	Left	2176	−8	3	60	20.59
IPL^1^	Right	933	52	−56	50	17.12
IPL^2^	Right	929	47	−30	48	15.41
MTG	Right	1598	51	−63	2	17.21
MTG	Left	747	−50	−72	10	14.11
SPL	Right	689	23	−59	43	14.70
LG	Right	1409	16	−78	−11	13.04
MOG	Right	1606	47	−80	10	14.51

*Note:* Regions, volumes, coordinates (xyz), and F‐statistics (F‐stat) reflect the peak voxel from the Montreal Neurological Institute atlas. Results were interpreted at *p* < 0.005_uncorrected_ (*p* < 0.05_FWEcorrected_). Cluster extent threshold: 467 mm^3^. Superscript numbers reflect separate areas of activation from the same anatomical region.

Abbreviations: dlPFC = dorsolateral prefrontal cortex; dmPFC = dorsomedial PFC; Hemi = hemisphere; IPL = inferior parietal lobule; LG = lingual gyrus; MOG = middle occipital gyrus; MTG = middle temporal gyrus; SPL = superior parietal lobe.

**Figure 2:**
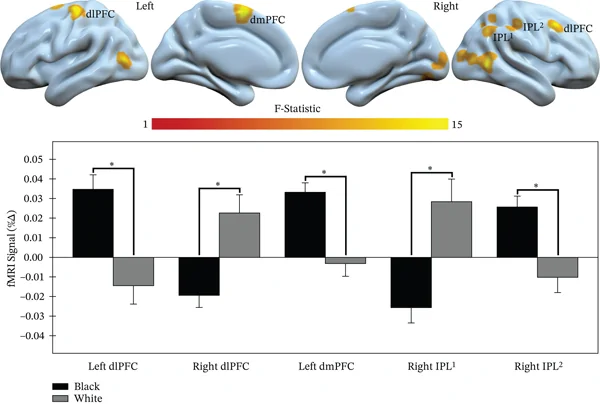
Race‐related differences in the neural response to stress. Note. Brain images (visualized in Surf Ice) depict the interaction between MIST condition (i.e., Control vs. Stress) and race (i.e., Black vs. White participants) from the LME analysis. Results were interpreted at *p* < 0.005_uncorrected_ (*p* < 0.05_FWEcorrected_). Cluster extent threshold: 467 mm^3^. Superscript numbers reflect separate areas of activation from the same anatomical region. Graphs (i.e., bar graphs) were used to visualize the race‐related differences that underlie the interaction (i.e., Race × MIST condition) from the LME analysis for a priori regions of interest. Specifically, bar graphs display stress‐elicited brain activity (i.e., extracted from significant a priori areas of activation identified by the LME analysis) during Control and Stress MIST conditions for Black and White participants. Bar graphs = mean value (Stress − Control MIST condition); error bars = standard error of the mean. Black participants (black bars) showed greater reactivity to stress than White participants (gray bars) within the left dorsolateral prefrontal cortex (dlPFC), left dorsomedial prefrontal cortex (dmPFC), and right inferior parietal lobule (IPL^2^). In contrast, White participants (gray bars) showed greater reactivity to stress than Black participants (black bars) within the right dlPFC and a separate area of the right IPL (IPL^1^). fMRI = functional magnetic resonance imaging; MIST = Montreal Imaging Stress Task; LME = linear mixed effects; FWE = family‐wise error.

Follow‐up analyses were then conducted to identify patterns of stress‐elicited brain activity that were uniquely associated with Black and White participants within the a priori regions of interest that showed a main effect of race (i.e., dmPFC, dlPFC, and IPL). Black participants showed greater reactivity to stress than White participants within the left dmPFC and dlPFC, as well as an area within the right IPL (Table 10; Figure 2). In contrast, Black participants showed lower reactivity to stress than White participants within the right dlPFC and a separate area of the right IPL (Table 10; Figure 2). These results demonstrate race‐related differences in stress reactivity within regions that underlie emotional expression, emotional regulation, and psychopathology [13].

**Table 10:** Race‐related comparisons of neural reactivity to stress.

Region	Hemi	Black	White	DF	*t*	*p* value
dlPFC	Left	0.03 (0.10)	−0.01 (0.09)	299	3.96	< 0.001^a^
dlPFC	Right	−0.02 (0.09)	0.02 (0.09)	299	−3.80	< 0.001^a^
dmPFC	Left	0.03 (0.07)	0.00 (0.06)	299	4.44	< 0.001^a^
IPL^1^	Right	−0.03 (0.11)	0.03 (0.12)	299	−3.92	< 0.001^a^
IPL^2^	Right	0.03 (0.08)	−0.01 (0.08)	299	3.76	< 0.001^a^

*Note:* Neural reactivity to stress reflects the differential response (i.e., Stress − Control) to the MIST. The values for Black and White reflect the mean (standard deviation). Analyses were performed for each a priori region of interest identified by the initial LME analysis as shown in Table 9. Multiple comparison corrections were completed using the Bonferroni correction for the five a priori regions of interest (0.05/5 = 0.01). Superscript numbers reflect separate areas of activation from the same anatomical region.

Abbreviations: DF = degrees of freedom; dlPFC = dorsolateral prefrontal cortex; dmPFC = dorsomedial prefrontal cortex; Hemi = hemisphere; IPL = inferior parietal lobule; LME = linear mixed effects; MIST = Montreal Imaging Stress Task; *t* = t‐statistic.

^a^Indicates the results survived Bonferroni correction.

#### 4.8.2. Relationship Between Neural Reactivity to Stress and ACEs

The LME analysis demonstrated a main effect of race for stress‐elicited brain activity within a priori regions of interest (i.e., dmPFC, dlPFC, and IPL) that support emotional expression and regulation (Tables 9 and 10; Figure 2). Significant differences in ACEs were also observed (Table 1). Therefore, we assessed whether the race‐related differences observed in stress‐elicited brain activity (as seen in Tables 9 and 10) also varied with ACEs.

**Figure 3:**
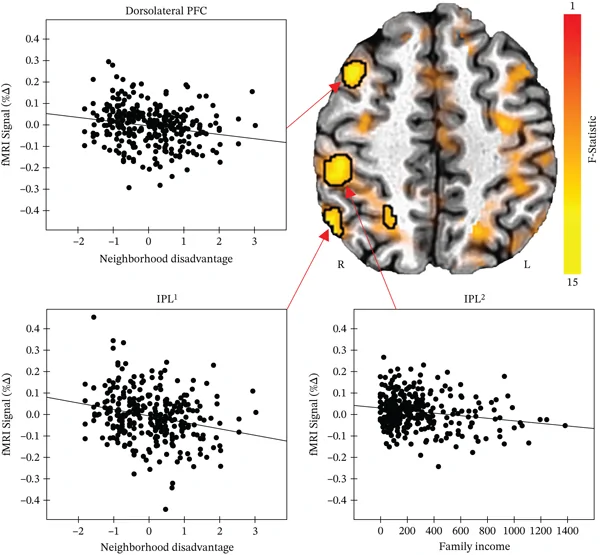
ACEs and the neural response to stress. Note. ACEs = adverse childhood experiences. The brain image (visualized in AFNI) depicts the interaction between MIST condition (i.e., Control vs. Stress) and race (i.e., Black vs. White participants) from the LME analysis. Results were interpreted at *p* < 0.005_uncorrected_ (*p* < 0.05_FWEcorrected_). Cluster extent threshold: 467 mm^3^. Suprathreshold regions of activation are depicted as fully opaque and outlined in black. Subthreshold activations are visible with decreasing opacity as statistical significance decreases. The graphs (i.e., scatterplots) depict data (Stress − Control MIST condition) extracted from significant a priori areas of activation. Neighborhood disadvantage was negatively related to stress‐elicited activity within the right dorsolateral prefrontal cortex (dlPFC) and right inferior parietal lobule (IPL^1^). Similarly, family income was negatively related to stress‐elicited activity in a separate area of the right IPL (IPL^2^). L = left hemisphere; R = right hemisphere; fMRI = functional magnetic resonance imaging; MIST = Montreal Imaging Stress Task; LME = linear mixed effects; FWE = family‐wise error.

Multiple linear regressions revealed significant relationships between ACEs and stress‐elicited brain activity (Table 11; Figure 3). Specifically, neighborhood disadvantage was negatively associated with right dlPFC and IPL activity (Table 11; Figure 3). Similarly, family income was negatively related to activity within a separate region of the right IPL (Table 11; Figure 3). No significant relationships were found with violence exposure (*p* > 0.05). However, these results suggest that the neural response to stress varies with ACEs in brain regions that support emotional processes [13].

**Table 11:** Functional regions of interest and ACEs.

Region	Hemi	Full model (F‐statistic)	*R* ^2^	Violence exposure *β*(*t*)	Neighborhood disadvantage *β*(*t*)	Family income *β*(*t*)
dlPFC	Left	2.74∗	0.04	−0.01 (−0.09)	0.03 (0.37)	−0.15 (−2.11)∗
dlPFC	Right	2.98∗	0.05	−0.01 (−0.20)	−0.20 (−2.81)∗∗^a^	−0.01 (−0.09)
dmPFC	Left	3.48∗∗^a^	0.06	−0.01 (−0.09)	0.05 (0.70)	−0.15 (−2.13)∗
IPL^1^	Right	4.25∗∗∗^a^	0.07	−0.06 (−0.96)	−0.26 (−3.68)∗∗∗^a^	−0.05 (−0.76)
IPL^2^	Right	3.30∗∗^a^	0.05	0.06 (0.96)	−0.04 (−0.63)	−0.20 (−2.77)∗∗^a^

*Note:* Neural reactivity to stress reflects the differential response (i.e., Stress − Control) to the MIST. Analyses were performed for each a priori region of interest identified by the initial LME analysis as shown in Table 9. Scanner and sex were included as covariates. Multiple comparison corrections were completed using the Bonferroni correction for the five a priori regions of interest (0.05/5 = 0.01). Superscript numbers reflect separate areas of activation from the same anatomical region.

Abbreviations: ACEs = adverse childhood experiences; dlPFC = dorsolateral prefrontal cortex; dmPFC = dorsomedial prefrontal cortex; Hemi = hemisphere; IPL = inferior parietal lobule; LME = linear mixed effects; MIST = Montreal Imaging Stress Task; *t* = t‐statistic; *β* = standardized beta coefficient.

^a^Indicates the results survived Bonferroni correction.

Significance: ∗*p* < 0.05; ∗∗*p* < 0.01; ∗∗∗*p* < 0.001.

### 4.9. ACEs Attenuate Race‐Related Differences in Neural Reactivity to Stress

Neuroimaging analyses revealed race‐related differences in stress‐elicited left dmPFC, bilateral dlPFC, and right IPL activity (Tables 9 and 10; Figure 2). Follow‐up analyses revealed relationships between ACEs (i.e., neighborhood disadvantage and family income) and stress‐elicited right dlPFC and right IPL activity (Table 11). Therefore, we investigated whether adjusting for ACEs would attenuate the race‐related differences in neural reactivity to stress. Effect sizes (Cohen′s *d*) were calculated to assess race‐related differences for the “raw” and “residual” models. The “raw” model only included covariates for scanner and sex, whereas the “residual” model included covariates for scanner, sex, and ACEs. Residual models included separate regressors for violence exposure, neighborhood disadvantage, and family income. The three ACEs were included in the same model because differences were observed between Black and White participants for all three ACEs (Table 1). Effect sizes were only calculated for a priori regions of interest that previously showed a main effect of race (i.e., dmPFC, dlPFC, and IPL; Tables 9 and 10; Figure 2).

#### 4.9.1. Effect Size Comparison

**Figure 4:**
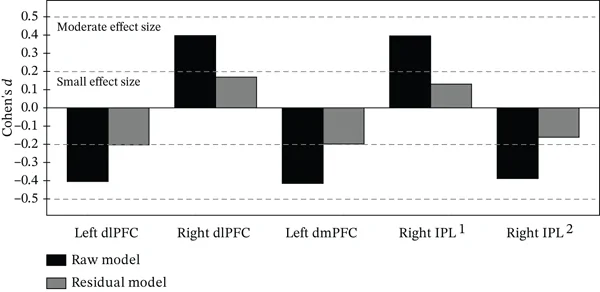
Effect sizes for race‐related differences in the neural response to stress. Note. Effect sizes (Cohen′s *d*) were calculated to assess race‐related differences (i.e., Black vs. White participants) in “raw” and “residual” neural reactivity to stress. The “raw” model only included covariates for scanner and sex, whereas the “residual” model included covariates for scanner, sex, and adverse childhood experiences (ACEs). Commonly accepted Cohen′s *d* guidelines were used to interpret effect sizes [81]. Positive Cohen′s *d* values reflect neural reactivity to stress that was greater in White than Black participants (i.e., White > Black), whereas negative Cohen′s *d* values reflect neural reactivity to stress that was greater in Black than White participants (i.e., Black > White). Moderate effect sizes were observed before adjusting for ACEs (“raw” model). However, race‐related differences were reduced to small effect sizes after ACEs were included in the model (“residual” model). dlPFC = dorsolateral prefrontal cortex; dmPFC = dorsomedial prefrontal cortex; IPL = inferior parietal lobule. Superscript numbers reflect separate areas of activation from the same anatomical region.

**Figure 5:**
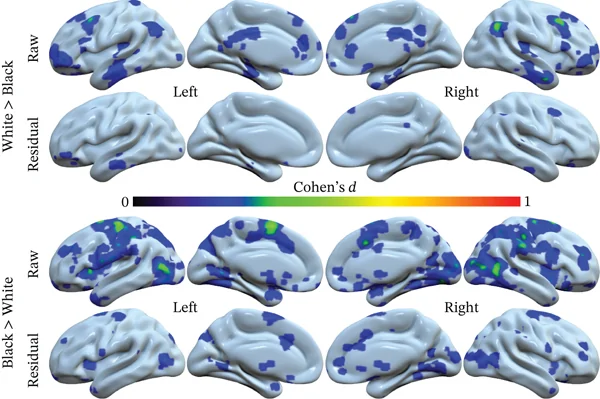
ACEs and race‐related differences in the neural response to stress. Note. ACEs = adverse childhood experiences. Brain images (visualized in Surf Ice) depict Cohen′s *d* values that were calculated to assess race‐related differences (i.e., Black vs. White participants) in “raw” and “residual” neural reactivity to stress. The “raw” model only included covariates for scanner and sex, whereas the “residual” model included covariates for scanner, sex, and ACEs. Cohen′s *d* values reflect neural reactivity to stress that was greater in White than Black participants (White > Black) and neural reactivity to stress that was greater in Black than White participants (Black > White). Moderate effect sizes were observed before adjusting for ACEs (“raw” model) for the dorsomedial prefrontal cortex, dorsolateral prefrontal cortex, and inferior parietal lobule. However, these race‐related differences were reduced to small effect sizes after ACEs were included in the model (“residual” model).

Race‐related differences in “raw” (i.e., only adjusting for covariates of scanner and sex) and “residual” (i.e., adjusting for covariates of scanner, sex, and ACEs) neural reactivity to stress were assessed using effect sizes (Cohen′s *d*). Commonly accepted Cohen′s *d* guidelines were used to interpret effect sizes [81]. Race‐related differences in the neural response to stress showed moderate effect sizes (0.39 [IPL^2^] to 0.42 [dmPFC]) before adjusting for ACEs (“raw” model) (Table 12; Figures 4 and 5). However, race‐related differences in the neural response to stress were reduced to small effect sizes (0.13 [IPL^1^] to 0.20 [left dlPFC]) after ACEs were included in the model (“residual” model) (Table 12; Figures 4 and 5).

**Table 12:** Neural reactivity to stress Cohen′s *d* values before and after adjusting for ACEs.

Model	dlPFC (L)	dlPFC (R)	dmPFC	IPL^1^	IPL^2^	Min	Max
Raw	0.40	0.40	0.42	0.40	0.39	0.39	0.42
Residual	0.20	0.17	0.20	0.13	0.16	0.13	0.20

*Note:* Effect sizes (Cohen′s *d*) were calculated to assess race‐related differences in “raw” and “residual” neural reactivity to stress. The “raw” model only included covariates for scanner and sex, whereas the “residual” model included covariates for scanner, sex, and ACEs. Cohen′s *d* values are expressed as absolute values. Superscript numbers reflect separate areas of activation from the same anatomical region.

Abbreviations: ACEs = adverse childhood experiences; dlPFC = dorsolateral prefrontal cortex; dmPFC = dorsomedial prefrontal cortex; IPL = inferior parietal lobule; L = left hemisphere; Max = maximum Cohen′s *d* value across a priori regions of interest that previously showed a main effect of race; Min = minimum Cohen′s *d* value across a priori regions of interest that previously showed a main effect of race; R = right hemisphere.

#### 4.9.2. Effect Size CI Comparison

**Figure 6:**
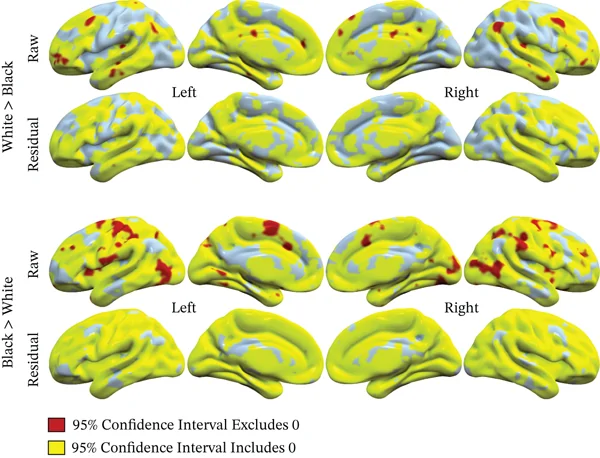
Confidence intervals for race‐related differences in the neural response to stress. Note. Brain images (visualized in Surf Ice) depict 95% confidence intervals (95% CIs) for “raw” and “residual” effect sizes (Cohen′s *d*). The “raw” model only included covariates for scanner and sex, whereas the “residual” model included covariates for scanner, sex, and adverse childhood experiences (ACEs). Cohen′s *d* values reflect neural reactivity to stress that was greater in White than Black participants (White > Black) and neural reactivity to stress that was greater in Black than White participants (Black > White). Cohen′s *d* values were confidently different from zero (i.e., 95% CIs excluded zero; red regions) before adjusting for ACEs (“raw” model) for the dorsomedial prefrontal cortex, dorsolateral prefrontal cortex, and inferior parietal lobule. However, Cohen′s *d* values were no longer confidently different from zero (i.e., 95% CIs included zero; yellow regions) after ACEs were included in the model (“residual” model).

95% CIs for the “raw” and “residual” models were calculated to determine whether the effect sizes (Cohen′s *d*) for each model confidently excluded zero (i.e., 95% CIs did not cross zero). Before adjusting for ACEs (“raw” model), 95% CIs for the dmPFC, dlPFC, and IPL did not cross zero (Table 13; Figure 6), suggesting that race‐related differences in the neural response to stress were different from zero. In contrast, after ACEs were included in the model, 95% CIs for these regions crossed zero, suggesting that race‐related differences in the neural response to stress were no longer different from zero (Table 13; Figure 6). Taken together, these findings suggest that ACEs may partially explain race‐related differences in the neural response to future stressors.

**Table 13:** Neural reactivity to stress Cohen′s *d* confidence intervals before and after adjusting for ACEs.

Model	dlPFC (L)	dlPFC (R)	dmPFC	IPL^1^	IPL^2^
Raw^a^	−0.646, −0.162	0.155, 0.639	−0.657, −0.173	0.153, 0.637	−0.630, −0.147
Residual	−0.444, 0.037	−0.072, 0.409	−0.439, 0.042	−0.109, 0.371	−0.401, 0.079

*Note:* 95% confidence intervals reflect effect sizes (Cohen′s *d*) from the “raw” and “residual” models. The “raw” model only included covariates for scanner and sex, whereas the “residual” model included covariates for scanner, sex, and ACEs. Confidence intervals are represented as “lower limit, upper limit.” Superscript numbers reflect separate areas of activation from the same anatomical region.

Abbreviations: ACEs = adverse childhood experiences; dlPFC = dorsolateral prefrontal cortex; dmPFC = dorsomedial prefrontal cortex; IPL = inferior parietal lobule; L = left hemisphere; R = right hemisphere.

^a^Confidence intervals exclude zero (i.e., do not cross zero) across all brain regions.

### 4.10. ACEs Mediate Race‐Related Differences in Stress Reactivity

Parallel mediation models revealed that race‐related differences in stress ratings and the neural response to stress were mediated by ACEs. These models (Table 14) reflect the combined mediation effect from all three ACEs (i.e., violence exposure, neighborhood disadvantage, and family income; total indirect effect) and individual mediation effects from each ACE. Similar to the residualization approach (see Table 8), the combined effect of all three ACEs fully mediated the relationship between participant race and stress ratings (Table 14). Moreover, this model revealed that each ACE independently mediated the relationship between race and stress ratings (Table 14). Individual mediation models (i.e., one mediator) were then used to understand to what extent each ACE contributed to the full mediation effect (Table 15). Violence exposure partially mediated the relationship between race and stress ratings (direct effect: *p* = 0.002), whereas neighborhood disadvantage (direct effect: *p* = 0.072) and family income (direct effect: *p* = 0.176) fully mediated this relationship (Table 15). Neighborhood disadvantage also fully mediated the relationship between participant race and stress‐elicited right IPL activity (i.e., IPL^1^) (Table 14). However, ACEs did not mediate the relationship between participant race and the remaining brain regions of interest (i.e., left dlPFC, right dlPFC, left dmPFC, and IPL^2^) (Table 14).

**Table 14:** Mediation: race, ACEs, and stress.

	Total effect		Direct effect		Total indirect	VE	ND	Income
	*β*	*p*	*β*	*p*	95% CI	95% CI	95% CI	95% CI
SR	−4.74	< 0.001	0.28	0.838	−6.96, −3.12^a^	−2.00, −0.22^a^	−3.42, −0.22^a^	−3.72, −0.56^a^
dlPFC (L)	0.05	0.001	0.05	0.004	−0.03, 0.02	−0.01, 0.01	−0.03, 0.01	−0.01, 0.03
dlPFC (R)	−0.04	< 0.001	−0.03	0.032	−0.03, 0.02	−0.01, 0.01	−0.03. 0.00	−0.01. 0.03
dmPFC	0.04	< 0.001	0.04	< 0.001	−0.02, 0.01	−0.01, 0.00	−0.02, 0.01	−0.01, 0.02
IPL^1^	−0.05	< 0.001	−0.04	0.072	−0.04, 0.01	−0.02, 0.01	−0.05, −0.01^a^	−0.00, 0.04
IPL^2^	0.04	< 0.001	0.03	0.016	−0.02, 0.03	−0.01, 0.01	−0.03, 0.00	−0.00, 0.03

*Note:* Parallel mediation models were constructed with participant race (X), ACEs (M) (i.e., violence exposure, neighborhood disadvantage, family income; adverse childhood experiences), and stress reactivity (Y) (i.e., stress ratings or neural response to stress). The total indirect effect reflects the combined mediation effect from all three ACEs. VE, ND, and Income reflect individual mediation effects. 95% confidence intervals (95% CI) are represented as “lower limit, upper limit.” Superscript numbers reflect separate areas of activation from the same anatomical region. Partial mediation was assumed if the direct effect remained significant after mediation, and full mediation was assumed if the direct effect did not remain significant after mediation [79].

Abbreviations: dlPFC = dorsolateral prefrontal cortex; dmPFC = dorsomedial prefrontal cortex; Income = family income; IPL = inferior parietal lobule; L = left hemisphere; ND = neighborhood disadvantage; R = right hemisphere; SR = stress ratings; VE = violence exposure; *β* = standardized beta‐coefficient.

^a^Confidence intervals exclude zero (i.e., do not cross zero), reflecting a significant mediation effect [79].

**Table 15:** Mediation: race, ACEs, and stress ratings.

Model	Total effect		Direct effect		Indirect effect
	*β*	*p*	*β*	*p*	95% CI
VE	−4.74	< 0.001	−3.45	0.002	−2.24, −0.43^a^
ND	−4.74	< 0.001	−2.22	0.072	−4.10, −0.95^a^
Income	−4.74	< 0.001	−1.70	0.176	−4.58, −1.51^a^

*Note:* Mediation models were constructed with participant race (X), ACEs (M; adverse childhood experiences), and stress ratings (Y). Violence exposure, neighborhood disadvantage, and family income reflect individual mediation models for each ACE. 95% confidence intervals (95% CI) are represented as “lower limit, upper limit.” Partial mediation was assumed if the direct effect remained significant after mediation, and full mediation was assumed if the direct effect did not remain significant after mediation [79].

Abbreviations: Income = family income; ND = neighborhood disadvantage; VE = violence exposure; *β* = standardized beta coefficient.

^a^Confidence intervals exclude zero (i.e., do not cross zero), reflecting a significant mediation effect [79].

## 5. Discussion

ACEs appear to have lasting effects on the neurobehavioral processes that underlie stress‐related emotional function [15–17, 82]. Due to systemic racial inequalities, exposure to adverse life experiences is typically greater for Black than White individuals [1, 3, 83]. Importantly, adverse life experiences may be particularly detrimental to the brain maturation and emotional development that occur during adolescence [4, 5, 84]. Thus, this investigation studied the interrelationship between race, ACEs, and neurobehavioral processes that support stress‐related emotional function. Paralleling previously published work, Black participants reported higher levels of violence exposure, greater neighborhood disadvantage, and lower family income than White participants [1, 3]. We also observed race‐related differences in the neurobehavioral response to stress. Further, the neurobehavioral response to stress varied with participant exposure to ACEs. In fact, adjusting for ACEs reduced the observed race‐related differences in participant responses to stress. These results suggest that ACEs may underlie race‐related differences in the neurobehavioral processes that support stress‐related emotional function.

Findings from this study demonstrate race‐related differences in stress‐elicited neurobehavioral processes, paralleling previously published work [45, 85, 86]. For example, prior Pavlovian fear conditioning research has described race‐related differences in threat‐elicited neural, behavioral, and psychophysiological responses [34, 86]. Similarly, the present study observed race‐related differences in neural (i.e., fMRI), behavioral (i.e., stress ratings), and psychophysiological (i.e., heart rate) responses to stress (Tables 5, 9, and 10; Figure 2). Pavlovian fear conditioning research has also suggested that the neurobehavioral response to threat varies with adverse life experiences [34]. Similarly, the present study found that participant stress ratings and neural reactivity to stress varied with ACEs (Tables 2, 7, and 11; Figure 3). Further, the race‐related differences in stress reactivity (i.e., stress ratings and brain activity) were reduced by including ACEs in the model (Tables 8, 12, and 13; Figures 4, 5, and 6). Thus, the observed race‐related differences in stress reactivity may be due, in part, to racial disparities in ACEs [34, 87]. Specifically, Black participants reported higher levels of violence exposure, greater neighborhood disadvantage, and lower family income than White participants (Table 1; Figure 1), consistent with the enduring systemic racial inequalities that underlie the current socioeconomic environment [1, 3, 88].

ACEs are closely linked to the development and expression of psychopathology [7, 9, 89, 90]. Consistent with prior research, the present study observed positive relationships between violence exposure and anxiety, depression, and posttraumatic stress (Table 2). However, the present study did not observe race‐related differences in these symptoms (Table 1). Thus, no race‐related differences in psychological distress were found, despite higher levels of violence exposure, greater neighborhood disadvantage, and lower family income among Black participants (Table 1). Given that prior work suggests that greater exposure to ACEs (as reported by Black participants) is linked to greater psychopathology [7–9], the failure to find a relationship between race and psychological distress in the present study may seem contradictory. However, other published work is consistent with this seemingly counterintuitive finding [91–94]. Specifically, in spite of greater exposure to adverse experiences, rates of psychopathology are often lower in Black than White individuals (i.e., “Black–White Mental Health Paradox”) [10, 11, 43, 95].

The lower prevalence of psychopathology often observed for Black individuals, despite greater exposure to ACEs (i.e., “Black–White Mental Health Paradox”), may reflect greater emotional resilience to adverse life experiences [10–12, 44]. Specifically, greater emotional resilience to adverse experiences may be a result of greater psychological preparedness towards adversity by those (e.g., Black individuals) who routinely face, and must overcome, systemic inequalities [10, 45, 46]. In contrast, White individuals who commonly experience less adversity may not develop the same level of emotional resilience as Black individuals [10, 45, 46]. For example, White individuals experience greater anxiety when witnessing violence against Black individuals due to their lack of experience with racially charged violence and racial discrimination [95, 96]. Prior research attributes the greater psychological preparedness (and lower psychopathology) exhibited by Black individuals to a higher sense of self‐esteem and greater access to coping resources (e.g., family, community, and religious support) [10, 12, 95, 97]. Additionally, Black individuals may develop a stronger social identity (i.e., greater racial identity) because of rejection (e.g., discrimination) from those outside of their social group (e.g., White individuals), often described as the “rejection‐identification model” [95, 98, 99]. In turn, a stronger racial identity protects Black individuals against psychopathology by offering a stronger sense of belonging, greater community support, higher self‐esteem, and race‐specific coping mechanisms [100–102].

Emotional resilience is supported by a distributed network of brain regions that include the amygdala, hippocampus, PFC, and IPL [18, 103–106]. The functional connectivity of these brain regions appears to be strengthened by early life adversity (e.g., stress acceleration hypothesis), which, in turn, supports more efficient emotion regulation, promoting resilience to future stress [107–110]. Systemic racial inequalities create conditions that increase the likelihood of ACEs among Black individuals relative to White individuals [1, 3, 88], including higher levels of violence exposure, greater neighborhood disadvantage, and lower family income (Table 1; Figure 1). Thus, ACEs may lead to changes in the function and connectivity of this neural circuitry, which, in turn, may underlie the emotional resilience of Black individuals to future stressors (i.e., “Black–White Mental Health Paradox”) [10, 11, 43, 95]. Importantly, each component of this neural circuit makes a unique, but interrelated, contribution to emotional resilience. For example, the hippocampus supports declarative memory processes that encode and interpret the significance of emotional material, which plays an important role in the contextualization of emotional experiences [29]. Hippocampal‐dependent memories then support cognitive reappraisal processes by shaping the interpretation of future stressors, which, in turn, modulates amygdala reactivity and the subsequent emotional response [18, 29, 111, 112]. Alongside hippocampal contributions, the frontoparietal network (i.e., PFC and IPL) supports higher order cognitive control processes (e.g., situational evaluation, emotional reframing, and shifting attention) that also modulate amygdala reactivity and emotional responses [18, 109, 113–115]. Thus, stronger functional integration between the amygdala, hippocampus, and frontoparietal network may underlie emotional resilience in those (e.g., Black individuals) with greater exposure, knowledge, and experience navigating adverse situations [10, 45, 46]. Moreover, the function of these brain regions (i.e., amygdala, hippocampus, PFC, and IPL) may also interact with psychosocial factors that promote emotional resilience (e.g., self‐esteem and social support) [100–102]. For example, greater self‐esteem and social support appear to strengthen amygdala–frontoparietal functional connectivity, which has been linked to more effective emotional regulation and reduced negative emotional reactivity [26, 116–119]. Although the present study did not study resilience directly, the observed neural activity may reflect coordinated interactions between cortical (i.e., PFC and IPL) and subcortical (i.e., amygdala and hippocampus) brain regions that support emotional resilience.

Differences in emotional resilience to adverse experiences may underlie the race‐related differences in stress‐elicited behavioral and psychophysiological responses that were observed in the present study. For example, prior research has found that Black individuals commonly consider chronic stressors as less upsetting than their White counterparts, which appears to parallel the lower rates of psychopathology observed in Black individuals [45, 46]. In the present study, stress ratings and heart rate to the MIST were lower for Black than White participants (Table 5). Interestingly, the race‐related differences in stress ratings were attenuated once ACEs were included in the model (Table 8). In fact, race‐related differences in stress ratings were reduced from high/moderate to small effect sizes, with CIs that crossed zero, suggesting that race‐related differences were substantially reduced and no longer different from zero (Table 8). Therefore, ACEs appear to contribute to race‐related differences in stress‐related behavior.

Prior exposure to adversity may promote processes (e.g., vigilance, habituation, and adaptation) that support resilience and explain the lower stress‐elicited response in those (i.e., Black individuals) exposed to greater adversity (see Tables 5 and 6). For example, those exposed to adversity often report heightened vigilance towards future stressors (e.g., monitoring surroundings and sensitivity to social cues) [120–122]. Although heightened vigilance may negatively impact mental health [121, 123], it may also enhance psychological preparedness among those who proactively attend to warning signals [124, 125]. For example, prior research has repeatedly found that predictable threats elicit smaller emotional responses than unpredictable threats [126–129]. Thus, greater vigilance may result in a larger anticipatory response, as an impending stressor is recognized, while the response to the stressor itself is reduced by the proactive use of emotion regulation strategies [130–132]. For example, ACEs in the present study were positively correlated with stress ratings during the Control condition of the MIST but negatively correlated with stress ratings during the Stress condition (Table 6). We also observed differences in stress reactivity between Black and White participants, consistent with ACE‐related vigilance. Specifically, Black participants, who had been exposed to more ACEs (Table 1), reported greater stress during the Control condition (i.e., greater baseline response) and lower stress during the Stress condition (i.e., blunted response to stress) than White participants (Table 16). Alternatively, these results may reflect stress‐related learning processes, in which individuals learn to regulate their responses to stress based on prior experience. For example, early and repeated stress exposure (e.g., adversity) progressively leads to smaller neural, hormonal, and behavioral stress responses (habituation hypothesis) [133–137]. Similarly, the knowledge gained from prior exposure to adversity may shape (adaptation hypothesis) future responses to stress [138–141]. Thus, prior exposure to adversity may modify emotional reactivity and/or regulation processes to support a more flexible stress response. This flexibility may present as heightened vigilance, followed by more rapid habituation, rather than sustained emotional hyperreactivity. Therefore, participants with greater exposure to ACEs may exhibit a combination of vigilance, habituation, and adaptation, ultimately resulting in greater emotional resilience and lower stress [10–12, 44].

**Table 16:** Race‐related differences in stress ratings to Control and Stress conditions of the MIST.

	Black	White	DF	*t*	*p* value
SR Control	15.82 (6.27)	14.02 (4.72)	245.54^a^	2.72	0.007
SR Stress	24.83 (7.02)	27.67 (5.91)	224.70^a^	3.62	< 0.001

*Note:* The values for Black and White participants reflect the mean (standard deviation) of stress ratings.

Abbreviations: DF = degrees of freedom; MIST = Montreal Imaging Stress Task; SR = stress ratings; SR Control = stress ratings for the Control MIST condition; SR Stress = stress ratings for the Stress MIST condition; *t* = t‐statistic.

^a^Adjusted for unequal variances.

ACEs also appear to underlie race‐related differences in the neural response to stress. Specifically, we found race‐related differences in stress‐elicited dmPFC, dlPFC, and IPL activity (Tables 9 and 10; Figure 2). Stress‐elicited activity within the dlPFC and IPL also varied with ACEs (i.e., neighborhood disadvantage and family income) (Table 11; Figure 3). Importantly, race‐related differences in stress‐elicited activity were reduced from moderate to small effect sizes after ACEs were included in the model (Tables 12 and 13; Figures 4, 5, and 6). Therefore, ACEs may also contribute to the race‐related differences in stress‐elicited neural activity observed in the present study (Tables 12 and 13; Figures 4, 5, and 6). The interrelationships among brain function, race, and ACEs were observed within brain regions that support emotional processes. For example, the frontoparietal network (i.e., dlPFC and IPL) supports the effortful regulation of emotion [22, 23, 142]. In fact, frontoparietal activation has been linked to greater emotional resilience to stress and lower psychopathology symptoms [18, 33]. Thus, exposure to adverse experiences during adolescence may have a lasting influence on the brain activity that supports emotional function.

Race‐related differences in dmPFC and dlPFC activity may partially explain the race‐related differences in stress‐elicited behaviors observed in this study. The dmPFC and dlPFC support processes that are important for emotion regulation [26, 143, 144], and dysfunction of these brain areas appears to disrupt stress‐related processes [6, 145–148]. However, the relationship between dorsal PFC activity, stress, and mental health may depend on which brain hemisphere (i.e., left or right) shows greater activity (often described as the “left–right imbalance hypothesis”). For example, greater activity in the left than right dlPFC is associated with a positive affect (e.g., greater emotion regulation, reduced sensitivity to aversive stimuli, and lower anxiety and depression symptoms) [149–152]. In contrast, greater activity in the right than left dlPFC is associated with a negative affect (e.g., reduced emotion regulation, greater behavioral inhibition, and greater sensitivity to aversive stimuli) [149–153]. Similar relationships have been observed within the dmPFC [154]. Further, transcranial magnetic stimulation (TMS) studies have found reductions in anxiety and depression symptoms linked to greater left dmPFC and dlPFC activity (high‐frequency TMS) and reduced right dmPFC and dlPFC activity (low‐frequency TMS) [155–160]. Thus, the “left–right imbalance hypothesis” may explain the race‐related differences in the dorsal PFC activity and stress‐elicited behaviors observed in this study. Specifically, Black participants showed greater left dmPFC and dlPFC activity, along with lower stress ratings and heart rate, than White participants (Tables 5 and 10; Figure 2). In contrast, White participants showed greater activity in the right dlPFC, along with greater stress ratings and heart rate, than Black participants (Tables 5 and 10; Figure 2). Although the “left–right imbalance hypothesis” may be consistent with the present results, further research is needed to strengthen the translation to fMRI. For example, support for this hypothesis is primarily derived from TMS and electroencephalogram studies [155–160], which may not translate directly into fMRI activation. Nevertheless, the greater left dmPFC and dlPFC activity parallels the lower stress reactivity of Black participants, whereas the right dlPFC activity parallels the greater stress reactivity of White participants.

Race‐related differences in IPL activity may further support the dorsal PFC′s (i.e., dmPFC and dlPFC) impact on stress reactivity (i.e., “left–right imbalance hypothesis”). Together, the PFC and IPL (as part of the frontoparietal network) work to regulate emotion [22, 23, 142]. Specifically, the dorsal PFC serves as the executive control center for emotion regulation (e.g., limiting attention to emotional triggers, reinterpreting emotional events, and inhibiting irrelevant responses), whereas the IPL supports the PFC via emotion‐related attentional control (e.g., encoding affective events, allocating attention toward or away from affective stimuli) [22, 23, 161–163]. Thus, the combined activity of the dorsal PFC and IPL appears to have important implications for healthy emotional function. For example, dual‐site stimulation (low‐frequency TMS) of both right dlPFC and IPL shows greater reductions in anxiety symptoms than stimulation to either location alone [164, 165]. The present study observed two distinct clusters of right IPL activation (Table 9; Figure 2). However, one IPL cluster showed greater activity for Black than White participants (i.e., IPL^2^), whereas the other cluster showed greater activity for White than Black participants (i.e., IPL^1^) (Table 10; Figure 2). This suggests that the IPL′s role as a support center for PFC‐facilitated emotional control may not show the same hemispheric patterns as the dmPFC and dlPFC. In fact, greater right IPL activity has been linked to both positive and negative affect, which differs from the left–right imbalance hypothesized for the dorsal PFC [149, 164, 166–168]. Thus, the right IPL activity observed for both Black and White participants (i.e., IPL^2^ and IPL^1^) in the present study may support the dorsal PFC as it modulates stress‐elicited behaviors. For example, IPL^1^ may work with the right dlPFC to enhance the stress reactivity of White participants, whereas IPL^2^ may work with the left dmPFC and dlPFC to lower the stress reactivity of Black participants. However, the IPL supports a broad range of cognitive functions (e.g., spatial attention, sensorimotor integration, theory of mind, and memory retrieval), and more work is needed to fully understand the IPL′s role in stress‐related emotion regulation [161, 169–171]. Similarly, the dorsal PFC plays a multifaceted role, integrating emotional valence processing with higher order functions such as cognitive control, working memory, attention regulation, response inhibition, and metacognitive monitoring [30, 172–175]. Future research designed to more fully characterize the function, laterality, and connectivity of this circuitry is needed to clarify the contributions of the IPL and PFC to race‐related and/or ACE‐related differences in emotional function.

The neural circuitry that supports stress‐related emotional processes includes the amygdala, hippocampus, PFC, and IPL [13, 14]. Therefore, we hypothesized that activity within these brain regions may vary with prior exposure to adversity and, thus, would show race‐related differences in the neural response to stress. However, we did not observe any race‐related differences in the amygdala or hippocampal response to stress, despite the use of small‐volume corrections. Importantly, the present study was focused on assessing race‐related differences in stress‐elicited brain activity. Thus, the lack of race‐related differences in the amygdala or hippocampus does not insinuate that these regions do not support stress‐related processes but rather that there are no differences between Black and White participants. In fact, prior research investigating social stimuli has observed similar amygdala reactivity for both Black and White individuals [176–178]. Given that systemic inequalities vary with race, the present findings suggest that the relationship between ACEs (and other race‐related socioenvironmental factors) and stress‐elicited brain activity may be stronger within regions that support higher level cognitive‐emotional processes (e.g., frontoparietal network) than those that support more automatic threat/arousal processes (e.g., amygdala and hippocampus). More specifically, although the amygdala, hippocampus, PFC, and IPL each support stress‐related processes [13, 14, 60, 179], the amygdala and hippocampus appear to support automatic and reflexive responses to stress that are mediated by the hypothalamic–pituitary–adrenal axis (HPA) and sympathetic nervous system [31, 32, 144, 180, 181]. In contrast, the frontoparietal network appears to support effortful and conscious emotional regulation processes, including resilience mechanisms suspected to underlie the “Black–White Mental Health Paradox” (e.g., limiting attention to emotional triggers, reinterpreting emotional events, and inhibiting irrelevant responses) [26, 143, 144]. Importantly, the frontoparietal network facilitates these strategies through the top‐down regulation of the amygdala and hippocampus [182, 183]. Thus, despite the pattern of activation observed in the present study, the coordinated function of this network may support unique and important aspects of the emotional response that ultimately underlie susceptibility and resilience to stress.

The present study used a residualization approach to assess whether race‐related differences in behavioral and neural responses to stress are related to ACEs. However, the residualization approach treats ACEs as regressors to statistically remove differences in lived experiences between racial groups, which may not adequately capture the complex processes (e.g., biological, social, and psychological) through which adversity shapes adolescent development [87, 184–186]. Therefore, we conducted exploratory parallel mediation analyses to assess whether ACEs mediate the relationship between race and stress reactivity. Similar to the initial residualization results (see Table 8), ACEs fully mediated the relationship between participant race and stress ratings (Table 14). Additionally, neighborhood disadvantage fully mediated the relationship between participant race and stress‐elicited right IPL activity (i.e., IPL^1^) (Table 14). These results suggest that neighborhood conditions (e.g., school quality, environmental hazards, and food access) play an important role in stress reactivity [187–189]. However, ACEs did not mediate the relationship between participant race and activity within the other a priori brain regions (i.e., PFC and IPL^2^; Table 14). Although the residualization approach is theoretically similar to mediation, there are methodological differences that may explain the inconsistent results. For example, the neural mediation models were constructed using extracted mean signal responses (AFNI: 3dROIstats), instead of the voxel‐based analysis used for the residualization approach. Furthermore, significant mediation effects require specific significant relationships between all variables of interest (i.e., X to Y, X to M, and M to Y). For example, the nonsignificant mediation effects (i.e., PFC and IPL^2^; Table 14) revealed significant race to neuroimaging (i.e., X to Y) and race to ACE effects (i.e., X to M). However, the ACE to neuroimaging effects (i.e., M to Y) were not significant. Importantly, mediation and residualization address related, but distinct, questions. More specifically, mediation assesses whether a third variable (M) explains the relationship between an independent (X) and dependent (Y) variable, whereas residualization assesses whether adjusting for a third variable reduces that relationship. In the present study, we hypothesized that race‐related differences in stress reactivity actually reflect differences in ACEs, given the well‐documented overlap between race and adverse exposures [87, 184–186]. Although the mediation models suggest that ACEs indirectly mediate the relationship between participant race and stress reactivity (see Tables 14 and 15), the residualization approach more directly demonstrates a reduction in race‐related differences after adjusting for ACEs (see Tables 12 and 13).

While lower stress reactivity among Black participants has often been interpreted as evidence of resilience (i.e., “Black–White Mental Health Paradox”) [10–12, 44], this pattern may reflect several distinct, but potentially overlapping, processes. For example, sustained exposure to adversity may also disrupt emotional function, leading to maladaptive outcomes, including the dysregulation of stress responsivity [186, 190, 191]. Specifically, repeated exposure to stress (e.g., systemic inequalities) may lead to the prolonged activation of stress‐related systems (e.g., sympathetic nervous system, endocrine system), which, in turn, may increase allostatic load [15, 190, 192]. Higher allostatic load has been linked to emotional dysregulation [193–195], which may present as a blunted emotional response [15, 196]. For example, prior exposure to adversity has been linked to a reduction in emotional reactivity (e.g., cortisol, cardiovascular, skin conductance) [196–201]. Thus, the lower stress reactivity observed among Black participants in the present study (Table 5), while generally consistent with ACE‐related resilience, may also reflect the repercussions of chronic stress exposure (e.g., allostatic load). Importantly, the literature linking ACEs with psychopathology has produced mixed results. While some prior work suggests that ACEs promote resilience, other work suggests that ACEs increase susceptibility to psychopathology [15, 202]. Thus, future research directly assessing mechanisms of resilience and susceptibility (e.g., coping, stress appraisal, and environmental factors) is needed to better understand the interrelationship between ACEs, neural development, and psychopathology.

The present findings complement a growing literature focused on the impact that ACEs have on brain function and stress‐related emotional processes [8, 15, 17, 68, 203]. Specifically, the ACEs assessed in the present study include dimensions of threat (e.g., violence exposure) and deprivation (e.g., neighborhood disadvantage and poverty) [34, 35, 204–206], which may have unique effects on neural and emotional development. However, the negative relationships we observed between these ACEs and stress reactivity also reflect a need for targeted intervention strategies, especially in populations exposed to greater amounts of adversity [1, 3]. For example, violence intervention strategies should focus on both reducing the prevalence of violence [207] and providing psychological support for adolescents who have been exposed to violence [208]. Correspondingly, neighborhood interventions may attempt to reduce psychological distress by targeting either economic composition (e.g., work support, wage supplements, and job guarantees) or residential status (e.g., housing assistance programs) [209–211]. Moreover, the negative impact of income inequality/poverty [38, 212] is often addressed through individualized (e.g., cash and loan assistance) or shared aid programs (e.g., energy subsidies, skill development, and nutrition support) [213–217]. However, broader socioenvironmental factors may reduce the success of these intervention programs. For example, prior work suggests that intervention approaches that provide resources and opportunities (e.g., targeting parental education and socioeconomic status) may not benefit the mental health (e.g., emotional resilience) of individuals from minority groups as much as their nonminority counterparts [218–222]. Therefore, intervention programs should both reduce ACEs and actively support minority populations to effectively leverage available resources.

## 6. Strengths and Limitations

The present study benefits from a large community sample (*N* = 301) from the Birmingham, Alabama metropolitan area. This region of the Southern United States is characterized by distinctive socioenvironmental features that remain relatively understudied in neuroscience research. However, the generalizability of the present findings may be limited, given that participants were drawn from a single geographic location. Likewise, the longitudinal data collection (i.e., 11–19 years of age), despite being a strength of the present study, may also influence the generalizability of our findings. Specifically, our final sample consists of participants who not only completed all four waves of data collection but were also willing and able to undergo neuroimaging. Moreover, although the original Healthy Passages study recruited participants without regard to race or ethnicity, the neuroimaging sample was restricted to Black and White participants in accordance with R01 funding priorities, which emphasized race‐related health behaviors in Black and White individuals. However, all eligible participants within these racial groups (who completed the Healthy Passages study) were equally recruited for neuroimaging. Despite these efforts, the possibility of unmeasured selection factors cannot be ruled out.

The present study operationalized participant race as a binary variable (i.e., Black and White), reflecting the racial groups of interests defined by the R01 funding mechanism that supported the original neuroimaging project. The present study then assessed whether racial differences in the neurobehavioral response to stress (i.e., between Black and White participants) are, in fact, a reflection of differences in adversity (i.e., violence exposure, neighborhood disadvantage, and family income). Although participant race was treated as a social construct reflecting differential exposure to systemic inequalities rather than a biological variable, our analytic approach utilized a categorical predictor, which certainly oversimplifies racial/ethnic heterogeneity. Furthermore, we would not expect a binary race variable to fully capture the complex sociocultural factors that buffer against and/or exacerbate the effects of ACEs (e.g., social support, religiosity, community factors, and institutional racism) [87, 184–186]. Although our statistical adjustment for ACEs may control for some pathways through which systemic racism affects neurodevelopment, it certainly does not capture all of them. Violence exposure, neighborhood disadvantage, and family income only represent a subset of life experiences relevant to neurodevelopment (e.g., religiosity, group identity, community engagement, family support and/or dysfunction, child maltreatment, and discrimination exposure). Moreover, ACEs often serve as proxies for broader clusters of correlated developmental influences (e.g., neighborhood disadvantage likely correlates with school quality, environmental hazards, and food access). Thus, the results of the present study should be interpreted with the understanding that there are many other socioenvironmental factors that likely influence race‐related differences. Nonetheless, the present study suggests that race‐related differences in stress reactivity reflect systemic inequality, and not inherent group characteristics.

Additional limitations involve the lack of longitudinal data for important variables of interest (i.e., brain function and psychological distress) and the use of two different MRI scanners. Although scanner was included as a covariate in all neuroimaging analyses, intrinsic differences between the MRI scanners (i.e., Allegra and Prisma) may introduce measurement variance and/or noise. Furthermore, the lack of longitudinal neuroimaging and behavioral data both precludes direct observation of neurodevelopmental trajectories and limits causal inferences about ACEs and neural development. For example, the observed brain activity could reflect early, recent, and/or ongoing adversity on brain development. Moreover, the observed brain activity may be influenced by other psychosocial and environmental factors that covary with the three ACEs assessed in this study. Future research would benefit from concurrent data collection across all variables of interest.

The interpretation of the cortisol results from the present study may be limited by methodological factors related to assessing cortisol in an MRI environment. More specifically, the evolution and recovery of the cortisol response are relatively slow compared to other psychophysiological measures (e.g., SCR, heart rate, and blood pressure). Introducing participants to the MRI environment often evokes stress prior to and independent of the stress task itself, as participants undergo safety screenings, receive warnings regarding potential hazards (e.g., metallic objects), and observe the precautions taken to ensure their safety [223]. In the present study, we observed elevated cortisol levels at baseline (i.e., pretask) and lower cortisol levels after the stress task (i.e., post‐task) (Table 3). Although stress research indicates that cortisol levels increase after a stressor [224, 225], prior MRI studies have obtained results similar to those observed in the current study [226, 227]. Specifically, each of these studies found greater cortisol levels prior to the MRI session, which has been attributed to anticipatory stress elicited by the MRI environment [223]. Importantly, high levels of pre‐MRI cortisol would, in turn, be expected to suppress further cortisol release (i.e., during the MRI stress task), which may lead to lower post‐task cortisol levels [223]. More specifically, following an elevation of cortisol levels, a negative feedback loop through the HPA axis inhibits the release of further cortisol [180, 181, 228]. However, other practicalities related to the collection of cortisol data may have also limited our ability to observe a traditional cortisol response. Specifically, participant and MRI scanner availability did not allow us to restrict cortisol collection to the afternoon, resulting in a large time window for data collection and an inability to control for diurnal rhythms, which likely introduced additional noise into the data. Therefore, the cortisol results should be interpreted with caution, and the data may not accurately reflect the cortisol response to the MIST under other conditions (e.g., outside the scanning environment).

## 7. Conclusion

This study investigated race‐related differences in the stress‐elicited neurobehavioral response. We observed race‐related differences in stress‐elicited behavior, psychophysiology, and brain function in regions that support emotional processes (i.e., dmPFC, dlPFC, and IPL). Additionally, we found that the neural response to stress varied with neighborhood disadvantage and family income, whereas the behavioral response to stress (i.e., stress ratings) varied with all three ACEs (i.e., violence exposure, neighborhood disadvantage, and family income). Importantly, adjusting for ACEs reduced race‐related differences in stress‐elicited responses. These findings suggest that race‐related differences in stress‐elicited emotional function reflect neurobiological consequences of systemic inequality, and not inherent group characteristics. The disproportionate exposure of ACEs among Black participants mirrors systemic racial inequalities found throughout the current socioeconomic environment. Thus, these findings provide insight into race‐related socioenvironmental factors that may underlie psychological/emotional function. Furthermore, this study highlights the importance of remaining vigilant about how the field interprets and communicates their findings.

## Funding

This study was supported by the National Institutes of Health, 10.13039/100000002, R01MH098348.

## Ethics Statement

The Healthy Passages study was approved by the Centers for Disease Control and Prevention and the original study site institutions [2, 50]. The current study was approved by the University of Alabama at Birmingham Institutional Review Board for Human Use [IRB‐120508007].

## Consent

All participants provided written informed consent as approved by the University of Alabama at Birmingham Institutional Review Board [IRB‐120508007].

## Conflicts of Interest

The authors declare no conflicts of interest.

## Data Availability

Participants in this study did not consent to the release of their data to a third party for reuse. Readers seeking access to the data should contact the corresponding author (David C. Knight). Data can only be released following completion of a data use agreement.
